# Protocol to investigate gene expression heterogeneity in cyanobacteria using mRNA CARD-FISH

**DOI:** 10.1016/j.xpro.2025.104212

**Published:** 2025-11-20

**Authors:** Anxhela Hania, Louison Dufour, Ondřej Prášil, Kasia Piwosz, Meri Eichner

**Affiliations:** 1 Laboratory of Photosynthesis, Institute of Microbiology of the Czech Academy of Sciences, Centre Algatech, Novohradská 237, 379 01 Třeboň, Czech Republic; 2 Faculty of Science, University of South Bohemia in České Budějovice, Branišovská 1645/31a, 370 05 České Budějovice, Czech Republic; 3 Department of Fisheries Oceanography and Marine Ecology, National Marine Fisheries Research Institute, Hugo Kołłątaja 1, 81 332 Gdynia, Poland

**Keywords:** Microbiology, Microscopy, Gene Expression, *In Situ* Hybridization, Environmental sciences

## Abstract

Here, we present a protocol for visualizing gene expression in the filamentous cyanobacterium *Trichodesmium* and the single-celled species *Synechocystis* and *Cyanothece* using the catalyzed reporter deposition fluorescence *in situ* hybridization (CARD-FISH) technique. We describe steps for fixation, agarose coating, enzymatic permeabilization, and sample handling. This protocol is broadly applicable to cyanobacteria, and the detection of *rbcL* mRNA in *Trichodesmium*, used as an example, supports its use to study the heterogeneity of physiological processes at single-cell level.

## Highlights

• Protocol for investigating heterogeneity of gene expression at single-cell level
• Instructions for permeabilization, coating, and sample handling
• Guidance for mRNA CARD-FISH protocol application in cyanobacteria

## Abstract (editor highlights)

Publisher’s note: Undertaking any experimental protocol requires adherence to local institutional guidelines for laboratory safety and ethics.

## Graphical abstract

**Figure:**
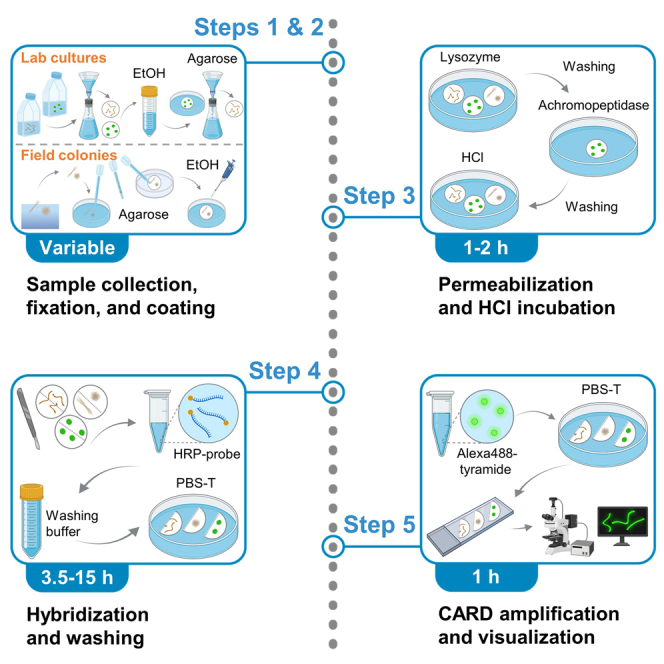

## Before you begin

### Innovation

This work introduces an optimized mRNA catalyzed reporter deposition fluorescence *in situ* hybridization (mRNA CARD-FISH) protocol for cyanobacteria. At first, we tested autofluorescence reduction and refined best fixation and permeabilization conditions in the filamentous, colony-forming *Trichodesmium*, as well as in the single-celled species *Synechocystis* and *Cyanothece*. To our knowledge, the comprehensive testing we performed has not previously been reported for cyanobacteria and revealed that fixation is the most crucial step for reliable results. In addition, we detailed practical handling tips for the fragile *Trichodesmium* filaments and colonies, which help to minimize filament fragmentation (a common but unreported issue) and colony loss during sample preparation and protocol execution. After confirming compatibility with natural sequence variability, we designed probe and helper oligonucleotides targeting an mRNA of interest in *Trichodesmium* as example of transcriptional detection. Unlike classical mRNA FISH, which requires multiple probes to achieve sufficient signal, CARD-FISH enables the visualization of cell-to-cell heterogeneity with a single probe. This technique thus provides an advantage for environmental samples, for which the design of multiple specific probes with similar hybridization profile would be challenging. Our finalized mRNA CARD-FISH protocol does not drastically differ from those generally used in the field, but the defined fixation step ensures its application in cyanobacteria, both in laboratory cultures and environmental samples.

### Background

CARD-FISH is a popular improvement of the FISH technique, widely used to study the composition, structure and function of complex microbial communities.^1^ FISH methods use oligonucleotide probes that bind to rRNA in ribosomes. The probes can be designed to be specific at various taxonomic levels, from species to phylum and even domain.^2^^,^^3^^,^^4^ They allow visualization of cells from distinct microbial lineages, and estimation of their absolute abundance in the samples. Yet, for visualizing less abundant mRNA, signal intensity is often too low. Hence, several approaches have been developed to enhance signal intensity (e.g., mRNA-FISH,^5^^,^^6^^,^^7^ mRNA CARD-FISH^8^^,^^9^^,^^10^^,^^11^). In FISH, the detection is achieved via 5′-fluorescently labeled probes,^12^ while in the CARD-FISH alternative, 5′-horseradish peroxidase (5′-HRP) labeled probes catalyze the deposition of fluorescently conjugated tyramide molecules at the site of hybridization.^13^^,^^14^ The resulting amplified signal generated by CARD-FISH, which is up to 200-fold brighter than FISH signals, allows the detection of almost inactive cells with low rRNA content and enables the detection of mRNA.^14^^,^^15^^,^^16^^,^^17^ A key advantage of CARD-FISH compared to other mRNA FISH methods is that it is enough to use a single probe, instead of multiple probes, allowing its use also in environmental samples for which it would be challenging to design a large number of probes. For these reasons, CARD-FISH has almost replaced the FISH technique in aquatic microbial ecology, especially in studies of oligotrophic environments. Several studies have exploited CARD-FISH protocols to detect specific mRNAs, such as *nifH* in N_2_-fixing bacteria,^8^ *nirS* in NO_2_^−^-reducing bacteria,^9^ and *mcyA* and *rpoB* in microcystin-producing cyanobacteria.^10^^,^^11^ All these studies primarily aimed to link specific functions to taxonomic groups for quantifying their abundance in environmental samples. The fact that mRNA CARD-FISH allows to address questions related to the regulation of physiological processes inspired us to optimize a protocol for visualizing gene expression patterns at the single-cell level in cyanobacterial cultures, as well as field samples.

Cyanobacteria are key organisms in the global carbon and nitrogen cycles, due to their dual role as primary producers and N_2_ fixers, and they hold great potential for biotechnological applications. The use of our optimized protocol can provide insights into the regulation of fundamental physiological processes in these organisms. However, the application of the mRNA CARD-FISH technique in cyanobacteria poses specific challenges due to their naturally low cellular mRNA content, the difficulty of effectively permeabilizing their membranes and cell walls, and finally their high autofluorescence.^18^^,^^19^^,^^20^^,^^21^^,^^22^^,^^23^ To overcome these issues, we adapted a standard CARD-FISH protocol^14^ for the transcript detection in three cyanobacterial species: the globally abundant filamentous genus *Trichodesmium* (including laboratory strains *T. erythraeum* IMS101 and NIBB1067, as well as field-collected colonies), and the two single-celled model species *Synechocystis* PCC 6803 (hereafter *Synechocystis*) and *Cyanothece* ATCC 51152 (hereafter *Cyanothece*). During the optimization process, we tested a wide range of fixation and permeabilization conditions, the efficiency of which was assessed based on the 16S rRNA-targeted probe EUB338-I to -III (hereafter EUB).^24^ Subsequently, the mRNA expression of the large subunit (*rbcL*) of ribulose-1,5-bisphosphate carboxylase/oxygenase (RubisCO) was inspected as an example of transcriptional detection on the laboratory cultures *T. erythraeum* IMS101 and NIBB1067 strains, as well as on field-collected colonies. Our analysis also includes negative control conditions, corresponding to samples exposed to NON-*rbcL* probe, NON-EUB probe,^25^ and to a hybridization solution lacking any probe (No-probe). Finally, we present a protocol broadly applicable to cyanobacteria, with guidance, hints and advice on the steps that might need refinement to ensure the reliability of mRNA CARD-FISH results.

### Probe and helper design

**Timing: variable**

***Note:*** Here, we based the probe sequence targeting *rbcL* mRNA for *Trichodesmium* laboratory cultures and field-collected colonies on the *rbcL* reverse primer sequence in Levitan et al.^26^ As the primer used in this study was specifically designed for *T. erythraeum* IMS101, we refined the probe sequence by performing an alignment of all *Trichodesmium rbcL* gene sequences available in the NCBI BioProject PRJNA804487.^27^ To account for the genetic variability among *Trichodesmium* natural populations, the final probe sequence includes degenerated oligonucleotide bases. The negative control NON-*rbcL* probe contains two middle mismatches compared to the *rbcL* probe. To enhance probe binding, we designed two unlabeled oligonucleotide helpers, A-*rbcL* and B-*rbcL*, to bind on the opposite loci of the target mRNA region. We based the helpers sequences on the top 20 structural outputs of the *rbcL* transcript (GenBank: AB075924.1) from the RNA structure prediction tool (https://rna.urmc.rochester.edu). Like the probes, the helper sequences also contain degenerated bases to cover natural sequence variability of *Trichodesmium* natural populations. All probe and helper details are summarized in Table 1.

***Note:*** For long molecules that may fold during translation, helpers can also be designed to bind the flanking regions of the probe target.

**Table 1:** List and sequences of the probes and helpers used in this study

Probe/Helper	Designation	Sequence (5′-3′)	Target	Description	Reference
Probe	*rbcL*	TCYTTVGCAAAMWCWGCACG	RubisCO large subunit	Gene of interest	This study
Probe	NON-*rbcL*	TCYTTVGCA**TT**MWCWGCACG	RubisCO large subunit	Negative control	This study
Probe	EUB	GCWGCCWCCCGTAGGWGT	16S rRNA	Positive control	Daims et al.^24^
Probe	NON-EUB	ACTCCTACGGGAGGCAGC	16S rRNA	Negative control, complementary for EUB	Wallner at al.^25^
Probe	CRY1-652	TTTCACAGTWAACGATCCGCGC	18S rRNA	Negative control used for the autofluorescence signal reduction test	Grujcic et al.^28^
Helper	A-*rbcL*	TCRTGCATRATGATTGG	RubisCO large subunit	Enhancer of *rbcL* probe site accessibility by binding to its predicted opposite loci	This study
Helper	B-*rbcL*	CGRCACCATYTSGCYARAGTTGT	RubisCO large subunit	Enhancer of *rbcL* probe site accessibility by binding to its predicted opposite loci	This study

Bold letters in the NON-*rbcL* sequence highlight the middle mismatches with respect to the *rbcL* sequence.

Y, M, W, R, S = degenerated bases.

### Preparation of fluorochrome solution

**Timing: 14 h**

1. Dissolve 1 mg (=1.6 μmol) of Alexa488 succinimidyl ester fluorochrome in 100 μL of N,N-dimethylformamide (see key resources table).

***Note:*** Esters are light sensitive and prone to hydrolysis. Hence, prepare them just before tyramide synthesis. Keep esters on ice until used for synthesis.

2. Add 25.2 μL of TYR-stock (see materials and equipment).
3. Incubate for 12 h at room temperature (RT = ∼20°C).
4. Add 874.8 μL of absolute ethanol (EtOH).
5. Make aliquots of 50 μL and store at −20°C (stable for 1 year). Alternatively, desiccate aliquots in a freeze dryer or under vacuum at RT, and store at −20°C (stable for years). For use, reconstitute with 50 μL of MilliQ water or N,N-dimethylformamide (containing 20 mg/mL of 4-iodophenylboronic acid, see key resources table), and store at −20°C.

***Note:*** 4-iodophenylboronic acid enhances the CARD-FISH signal.^29^ Hence, the second reconstitution option is preferable.

### Preparation of paraformaldehyde (PFA) solution

**Timing: 2.5 h**

***Note:*** We did not employ PFA in our final protocol, but we describe its preparation to account for its use during the testing phase (see fixation and permeabilization tests).

**CRITICAL:** PFA (see key resources table) is a toxic compound, hence, perform all steps of its preparation and its use in a fume hood.

6. Into a beaker, add 70 mL of MilliQ water, and heat to 60°C.
7. Depending on the desired concentration, add 20 g (20%), 15 g (15%), or 10 g (10%) of PFA. Heat and stir for approximately an hour (PFA dissolution is slow).

**CRITICAL:** Temperature should not exceed 60°C, otherwise PFA might burn and the preparation must be repeated.

8. Add a few drops of NaOH 1 M to complete the dissolution of all powder.
9. When the powder is completely dissolved, add 10 mL 10 × PBS.
10. Check and adjust pH as needed (7.2 in here), and bring the volume up to 100 mL with MilliQ water.
11. Cool down to 4°C.
12. Filter the solution.
13. Store at −20°C (for several weeks) or 4°C (for several days).

**CRITICAL:** Prioritize freshly prepared PFA solution as much as possible. Do not refreeze it after defrosting.

***Note:*** For *Trichodesmium* colonies, which were sampled and fixed in the field, 8% PFA aqueous solution EM Grade was diluted with either 0.2-μm-filtered seawater from the sampling site or with PBS to the respective final concentration.

### Autofluorescence signal reduction test

***Note:*** One important step in application of CARD-FISH to photosynthetic organisms is to ensure proper reduction of autofluorescence signal to avoid interference with the signal of the fluorescent label. Here, we verified autofluorescence signal reduction in the phycobiliprotein region on filtered *T. erythraeum* NIBB1067 laboratory cultures by testing more than ten different chemical treatments:

 15 min 96% ethanol (EtOH).

 15 min 100% methanol (MetOH).

 15 min mixture of 90% acetone and 100% MetOH (7:2, vol/vol; this treatment damaged the filter and, thus, was not further processed).

 15 min 0.1% H_2_O_2_.

 1 h 1% sodium dodecyl sulfate (SDS).

 1 h 1% SDS + 1 h 1% H_2_O_2_.

 1 h 1% SDS + 1 h 2% H_2_O_2_.

 1 h 1% SDS + 1 h 3% H_2_O_2_.

 24 h 100% MetOH.

 1 h 1% SDS + 24 h 100% MetOH.

 1 h 1% SDS + 1 h 1% H_2_O_2_ + 24 h 100% MetOH.

 1 h 1% SDS + 1 h 2% H_2_O_2_ + 24 h 100% MetOH.

 1 h 1% SDS + 1 h 3% H_2_O_2_ + 24 h 100% MetOH.

**Figure 1:**
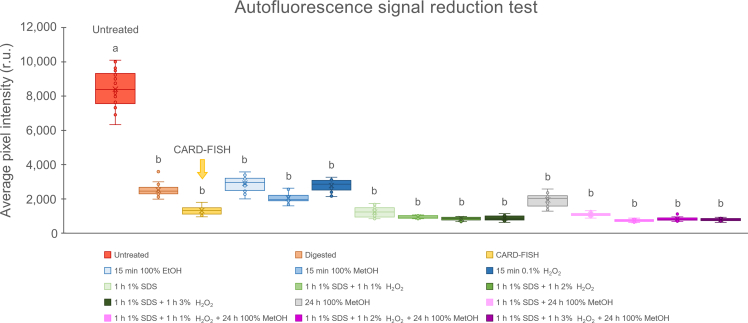
Average pixel intensity of phycobiliprotein region autofluorescence measured after different treatments to test signal reduction The results indicate that the standard CARD-FISH treatment (yellow box) alone is sufficient to substantially reduce the autofluorescence signal. Each treated sample is compared to the untreated sample. Different letters above the boxes denote statistically significant differences (Wilcoxon Rank Sum test, *p* = 0.05). Error bars represent the standard deviation of mean intensity values obtained from 1 to 3 filaments, each comprising measurements (performed with the circle tool in ZEN.blue software) from 19 to 26 circles. r.u. = relative unit; EtOH = ethanol; MetOH = methanol; SDS = sodium dodecyl sulfate.

We conducted all above-mentioned incubations at RT, after dipping filters in 0.15% warm agarose, digesting them with lysozyme for 1 h at 37°C and incubating them with 0.01 M HCl for 10 min at RT (as described in the step-by-step method details). We compared the autofluorescence of all samples to that of untreated sample (only filtered), digested sample (agarose + lysozyme + HCl) and standard CARD-FISH sample (agarose + lysozyme + HCl + CRY1-652,^28^ see probe details in Table 1). We detected autofluorescence with the epifluorescence microscope (see key resources table) using Alexa546 settings (see protocol step-by-step method details for more details) and an exposure time of 2.86 ms (note that the exposure time was adjusted for the untreated sample and kept constant afterward). We measured the average pixel intensity for an area with the circle tool in ZEN.blue software (see key resources table). We analyzed around 19 to 26 circles from 1 to 3 filaments. We conducted statistical analysis in R (R Core Team, https://www.R-project.org). Specifically, we assessed data normality using the Shapiro-Wilk test (shapiro.test) and evaluated the differences of each treatment to the untreated sample using the Wilcoxon’s Rank Sum test (wilcox.test), with significance threshold set to *p* = 0.05. All tested treatments lowered autofluorescence compared to the untreated sample (Figure 1). The most considerable decrease occurred when using SDS alone or in combination with H_2_O_2_ and H_2_O_2_ + MetOH. We also observed a strong decrease in autofluorescence after the standard CARD-FISH treatment alone (i.e., agarose + lysozyme + HCl + CRY1-652). Based on this observation, we considered the standard treatment sufficiently effective to achieve a substantial reduction in autofluorescence levels and, consequently, none of the tested chemical treatments were deemed necessary. Overall, such finding highlights the robustness and effectiveness of the CARD-FISH method for minimizing autofluorescence.

### Fixation and permeabilization tests

***Note:*** The CARD-FISH method includes the following steps: sample collection, fixation, coating, permeabilization, hybridization, washing, catalyzed reporter deposition (CARD, also referred to as tyramide signal amplification, TSA), and visualization.^30^ To determine the optimal protocol conditions for our three cyanobacteria species, we performed a series of tests including: various incubation times in EtOH, PFA, and GA (see key resources table) for fixation; different combinations and incubation times in lysozyme and achromopeptidase for permeabilization; and finally, several concentrations and incubation times of EDTA for permeabilization. During these optimization trials (listed in Table 2), permeabilization efficiency was assessed based on the 16S rRNA targeted probe EUB.

***Note:*** Hereafter, we present the finalized step-by-step method details protocol validated in *Trichodesmium*, *Synechocystis*, and *Cyanothece*, as well as its application for investigating mRNA transcriptional heterogeneity using *rbcL* as an example. We tested the expression patterns of *rbcL* mRNA (for identifying best hybridization conditions) and visualized this gene in *Trichodesmium* laboratory strains and natural colonies, using oligonucleotide helpers to enhance accessibility of the probe site. In these experiments, we used EUB as a positive control while NON-*rbcL*, NON-EUB, and No-probe (Table 1) served as negative controls.

**Table 2:** List of the performed tests to achieve optimal protocol conditions

Fixation	EDTA	Lysozyme	Achromopeptidase	Optimal conditions for
96% EtOH, overnight, −20°C	–	–	–	–
96% EtOH, 1 h, −20°C	–	1 h	–	–
96% EtOH, 1 h, −20°C	–	1 h	30 min	–
0.5% GA, 5 min, 4°C 96% EtOH, 50% EtOH, each 10 min, 4°C	–	1 h	–	–
0.5% GA, 5 min, 4°C 96% EtOH, overnight, −20°C	–	1 h	–	–
1.5% GA, 5 min, 4°C 96% EtOH, 50% EtOH, each 10 min, 4°C	–	1 h	–	–
1.5% GA, 5 min, 4°C 96% EtOH, overnight, −20°C	–	1 h	–	–
96% EtOH, overnight, −20°C 4% PFA in PBS, 30 min, RT	–	–	–	–
96% EtOH, overnight, −20°C 4% PFA in PBS, 30 min, RT	–	1 h	–	–
0.5% PFA in PBS, 5 min, 4°C 96% EtOH, 50% EtOH, each 10 min, 4°C	–	1 h	–	–
0.5% PFA in PBS, 5 min, 4°C 96% EtOH, overnight, 4°C	–	1 h	–	–
1.5% PFA in PBS, 5 min, 4°C 96% EtOH, 50% EtOH, each 10 min, 4°C	–	1 h	–	–
1.5% PFA in PBS, 5 min, 4°C 96% EtOH, overnight, 4°C	–	1 h	–	–
1.5% PFA in PBS, 10 min, 4°C 96% EtOH, overnight, 4°C	–	1 h	–	–
1.5% PFA in PBS, 30 min, 4°C 96% EtOH, overnight, 4°C	–	1 h	–	–
96% EtOH, overnight, −20°C	–	1 h	–	*Trichodesmium*
96% EtOH, overnight, −20°C	–	1 h	15 min	–
96% EtOH, overnight, −20°C	–	1 h	30 min	*Cyanothece*
96% EtOH, overnight, −20°C	–	1 h	1 h	*Synechocystis*
96% EtOH, overnight, −20°C	–	1 h	2 h	–
4% PFA in PBS, 1 h, 4°C	–	1 h	–	–
4% PFA in PBS, 1 h, 4°C	–	1 h	30 min	–
4% PFA in PBS, 1 h, 4°C	–	1 h	2 h	–
4% PFA in PBS, overnight, 4°C	–	1 h	–	–
4% PFA in PBS, overnight, 4°C	–	2 h	30 min	–
4% PFA in PBS, overnight, 4°C	–	1 h	30 min	–
4% PFA in SW, overnight, 4°C	–	1 h	30 min	–
4% PFA in PBS, overnight, 4°C	–	1 h	1 h	–
4% PFA in PBS, overnight, 4°C	–	1 h	2 h	–
4% PFA in SW, overnight, 4°C	–	2 h	30 min	–
2% PFA in SW, overnight, RT	–	1 h	1 h	–
2% PFA in SW, overnight, RT	–	1 h	2 h	–
4% PFA in PBS, 22 h, 4°C	–	1 h	2.5 h	–
2% PFA in SW, 22 h, 4°C	–	1 h	3 h	–
4% PFA in PBS, 22 h, 4°C	10 mM, 30 min	1 h	2 h	–
2% PFA in SW, overnight, RT	10 mM, 1.5 h	1 h	2 h	–
4% PFA in PBS, 25 h, 4°C	50 mM, 30 min	1 h	2 h	–
4% PFA in PBS, 25 h, 4°C	100 mM, 30 min	1 h	2 h	–

Each row represents a single test in which incubations were applied sequentially.

EtOH, ethanol; GA, glutaraldehyde; PFA, paraformaldehyde; % EtOH, final concentration of ethanol; % GA, final concentration of glutaraldehyde; % PFA, final concentration of PFA; PBS, 1 × PBS; SW, sea water; RT, room temperature.

## Key resources table

**Table:** 

REAGENT or RESOURCE	SOURCE	IDENTIFIER
**Chemicals, peptides, and recombinant proteins**		

EtOH	VWR	Cat#20816.298
PFA	Sigma-Aldrich	Cat#158127-100G
8% PFA aqueous solution EM grade	Electron Microscopy Sciences	Cat#157-8
GA	Sigma-Aldrich	Cat#104239
Lysozyme	PanReac ApliChem	Cat#A3711,0010
Achromopeptidase	Sigma-Aldrich	Cat#A3547-100KU
HCl	Penta	Cat#84421M1000
NaCl	Lach-Ner	Cat#30093-AP0-G1000-1
KCl	Lach-Ner	Cat#30076-AP0-G1000-1
Na_2_HPO_4_ · 12H_2_O	Penta	Cat#04273G1000
KH_2_PO_4_	Lach-Ner	Cat#30145
Tris	Invitrogen	Cat#15-506-017
EDTA	PanReac ApliChem	Cat#131669,1209
SDS	Sigma-Aldrich	Cat#71725-100G
Agarose	BioConcept	Cat#7-01P02-O
Triton X-100	Sigma-Aldrich	Cat#93443-10ML
H_2_O_2_	Sigma-Aldrich	Cat#386790-100ML
Dextran sulfate	Sigma-Aldrich	Cat#D8906-100G
Blocking reagent	Roche Diagnostics	Cat#11096176001
Maleic acid	Glentham Life Sciences	Cat#GK3899
Formamide	Sigma-Aldrich	Cat#47671-25ML-F
N,N-dimethylformamide	Sigma-Aldrich	Cat#D4551-250ML
4-iodophenylboronic acid	Sigma-Aldrich	Cat#471933-25G
Glycerol	Electron Microscopy Sciences	Cat#17970-25
VECTASHIELD	BIOZOL	Cat#H-1000
DAPI	Sigma-Aldrich	Cat#D9542-1MG
Tyramine-HCl	Sigma-Aldrich	Cat#T2879-1G
Triethylamine	Sigma-Aldrich	Cat#90335-100ML
Fluorochrome Alexa488 succinimidyl ester	Invitrogen	Cat#A20000

**Oligonucleotides**		

HRP-labeled probe CRY1-652	Biomers.net	https://biomers.net/
HRP-labeled probe EUB338-I to -III	Biomers.net	https://biomers.net/
HRP-labeled probe NON-EUB	Biomers.net	https://biomers.net/
HRP-labeled probe *rbcL*	Biomers.net	https://biomers.net/
HRP-labeled probe NON-*rbcL*	Biomers.net	https://biomers.net/
Helper A-*rbcL*	Generi Biotech	https://www.generi-biotech.com/
Helper B-*rbcL*	Generi Biotech	https://www.generi-biotech.com/

**Softwares and algorithms**		

ZEN.blue (v.2.3)	Carl Zeiss Microimaging	N/A
ZEN.black (v.2.3SP1)	Carl Zeiss Microimaging	N/A
ImageJ (v.1.54g)	Schneider et al.^31^	N/A

**Other**		

Polycarbonate filters, 47 mm, 5 μm	Whatman	Cat#WHA70604713
Polycarbonate filters, 47 mm, 0.4 μm	Whatman	Cat#WHA10417712
Polycarbonate filters, 25 mm, 0.8 μm	Whatman	Cat#WHA10417306
Glass slides	VWR	Cat#631-1553
Regular cover slips	VWR	Cat#630-2103
High-precision cover slips	Electron Microscopy Sciences	Cat#71861-054
Filtration device	Rocker	Cat#MF30
Sedgewick Rafter counting chamber	Graticules Optics	Cat#02C00415
Automatic cell counter	Beckman Coulter	Model#Multisizer4
Hybridization oven	UVP	Model#HM-4000 Multidizer
Epifluorescence microscope	Carl Zeiss Microscopy	Model#Zeiss Axio Imager.Z2
Confocal microscope	Carl Zeiss Microscopy	Model#Zeiss LSM 880

## Materials and equipment

**Table:** 0.15% Agarose

Reagent	Final concentration	Amount
Agarose	0.15%	0.3 g
MilliQ water	N/A	200 mL
**Total**	**N/A**	**200 mL**

Using a microwave, heat until agarose is completely dissolved. Store the solution at RT for up to several months. Melt each time before use.

**Table:** 10 × PBS

Reagent	Final concentration	Amount
NaCl	1.37 M	40 g
KCl	27 mM	1 g
Na_2_HPO_4_ · 12H_2_O	101 mM	18.15 g
KH_2_PO_4_	17.6 mM	1.2 g
MilliQ water	N/A	to 500 mL
**Total**	**N/A**	**500 mL**

Adjust pH to 7.4, autoclave, and store the solution at RT for up to a month. The 1 × PBS solution can be quickly prepared from the 10 × PBS stock solution when needed (e.g., during the washing steps, see step 5e step-by-step method details).

**Table:** 30 KU Achromopeptidase stock

Reagent	Final concentration	Amount
Achromopeptidase (100 KU)	30 KU	40 mg
MilliQ water	N/A	3.3 mL
**Total**	**N/A**	**3.3 mL**

Make aliquots and store them at −20°C for up to several months.

**Table:** TRIS-HCl pH 7.4 & pH 8

Reagent	Final concentration	Amount
TRIS	1 M	15.76 g
MilliQ water	N/A	to 100 mL
**Total**	**N/A**	**100 mL**

Prepare two solutions of TRIS-HCl and adjust pH to 7.4 and 8, respectively. Autoclave and store the solutions at RT for up to several months.

**Table:** 5 M NaCl

Reagent	Final concentration	Amount
NaCl	5 M	116.884 g
MilliQ water	N/A	to 400 mL
**Total**	**N/A**	**400 mL**

Autoclave and store the solution at RT for up to a year.

**Table:** NaCl-TRIS buffer

Reagent	Final concentration	Amount
NaCl (5 M)	10 mM	400 μL
TRIS-HCl (1 M, pH 8)	10 mM	2 mL
MilliQ water	N/A	to 200 mL
**Total**	**N/A**	**200 mL**

Adjust pH to 8, sterilize by filtration on 0.2 μm syringe filter, and store the solution at RT for up to several months.

**Table:** 0.5 M EDTA

Reagent	Final concentration	Amount
EDTA	0.5 M	14.612 g
MilliQ water	N/A	to 100 mL
**Total**	**N/A**	**100 mL**

Stir until EDTA powder is completely dissolved, autoclave, and store the solution at RT for up to several months. Note that EDTA dissolves better when adjusting pH to 8.

**Table:** 10% SDS

Reagent	Final concentration	Amount
SDS	10%	1 g
MilliQ water	N/A	to 10 mL
**Total**	**N/A**	**10 mL**

Dissolve at 40°C and store the solution at RT for up to several months.

**Table:** 0.01% PBS-T

Reagent	Final concentration	Amount
10 × PBS	1 ×	50 mL
Triton X-100 (10%)	0.01%	500 μL
MilliQ water	N/A	to 500 mL
**Total**	**N/A**	**500 mL**

Store the solution at RT for up to several months.

**Table:** 0.15% H_2_O_2_

Reagent	Final concentration	Amount
30% H_2_O_2_	0.15%	5 μL
MilliQ water (or 1 × PBS)	N/A	1 mL
**Total**	**N/A**	**1.005 mL**

Store the solution at 4°C for up to 3 weeks.

**Table:** 10% Blocking reagent

Reagent	Final concentration	Amount
NaCl	0.15 M	0.438 g
Maleic acid	0.1 M	0.5805 g
Blocking reagent	10%	5 g
MIlliQ water	N/A	to 50 mL
**Total**	**N/A**	**50 mL**

Make aliquots and store them at −20°C for up to several months.

***Note:*** Initially, add only few mL of MilliQ water (5–10 mL) and adjust pH to 7.5 with NaOH (1 M, ∼50–150 μL). Monitor pH while stirring and heating until blocking reagent is completely dissolved, then successively bring the volume up to 50 mL.

**Table:** Amplification buffer

Reagent	Final concentration	Amount
10 × PBS	1 ×	4 mL
NaCl (5 M)	2 M	16 mL
Dextran sulfate	10%	4 g
MilliQ water	N/A	to 40 mL
10% Blocking reagent	0.1%	400 μL
**Total**	**N/A**	**40 mL**

Store the solution at 4°C for up to several weeks.

***Note:*** Because dextran sulfate is a sticky compound, prepare this solution in a 50 mL Falcon tube. After adding dextran sulfate, dissolve it by rotation in a heated oven (∼40°C). Cool down to 4°C (to avoid precipitation of blocking reagent) and then add the remaining components at RT.

**Table:** Hybridization buffer (% HB)

Reagent	Final concentration	Amount
NaCl (5 M)	0.9 M	3.6 mL
TRIS-HCl (1 M, pH 7.4)	0.02 M	400 μL
Dextran sulfate	10%	2 g
Formamide	Variable	X mL (see table below)
MilliQ water	N/A	X mL (see table below)
10% Blocking reagent	1%	2 mL
10% SDS	0.01%	20 μL
**Total**	**N/A**	**20 mL**

Make aliquots and store them at −20°C for up to a year.

***Note:*** Because dextran sulfate is a sticky compound, prepare this solution in a 50 mL Falcon tube. After adding dextran sulfate, dissolve it by rotation in a heated oven (∼40°C). Cool down to 4°C (to avoid precipitation of blocking reagent) and then add the remaining components at RT.

**Table:** Amount of formamide and MIlliQ water for the preparation of the hybridization buffer (% HB)

% HB	mL formamide	mL MilliQ water
20	4	10
25	5	9
30	6	8
35	7	7
40	8	6
45	9	5
50	10	4
55	11	3
60	12	2
65	13	1
70	14	0

**Table:** DAPI

Reagent	Final concentration	Amount
DAPI	100 μg/mL	1 mg
MilliQ water	N/A	10 mL
**Total**	**N/A**	**10 mL**

Make aliquots and store them at −20°C for up to a year.

**Table:** Antifading mounting mix

Reagent	Final concentration	Amount
Glycerol	70.7% vol/vol	5 mL
Vectashield	14.1% vol/vol	1 mL
10 × PBS	1.4 ×	1 mL
DAPI (100 μg/mL)	1 μg/mL	70 μL
**Total**	**N/A**	**7.070 mL**

Make aliquots and store them at 4°C in the dark for up to several months.

***Note:*** DAPI can be omitted if not needed.

**Table:** TYR-stock

Reagent	Final concentration	Amount
N,N-dimethylformamide	99% vol/vol	1 mL
Triethylamine	0.99% vol/vol	10 μL
Tyramine-HCl	9.90 mg/mL	10 mg
**Total**	**N/A**	**1.010 mL**

Make aliquots of 100 μL (=5.76 μmol) and store them at −20°C for up to several months.

**Table:** Probes

Reagent	Final concentration	Amount
HRP-labeled probe	50 ng/μL	Depends on the stock provided by the supplier
MilliQ water	N/A	Depends on HRP-labeled probe volume
**Total**	**N/A**	**Variable**

Make aliquots of 20–50 μL and store the one currently in use at 4°C for up to several months. Other aliquots should be kept at −20°C. Frequent thawing and freezing of the probe may result in HRP dissociation.

**Table:** Helpers

Reagent	Final concentration	Amount
Helper	50 ng/μL	Depends on the stock provided by the supplier
MilliQ water	N/A	Depends on helper volume
**Total**	**N/A**	**Variable**

Make aliquots and store them at −20°C for up to a year.

## Step-by-step method details

### Sample collection

**Timing: 10 min–variable**

**Timing: 10 min (for step 1)**

**Timing: variable (for step 2)**

Here, we describe steps for the sample collection of *Trichodesmium*, *Synechocystis*, and *Cyanothece* laboratory cultures and *Trichodesmium* field colonies.

1. Sample collection of *Trichodesmium*, *Synechocystis*, and *Cyanothece* laboratory cultures.
  ***Note:*** Sample collection of *Trichodesmium*, *Synechocystis* and *Cyanothece* laboratory cultures is schematically illustrated in in Figures 2Ai and iii.
  a. Collect laboratory cultures by filtration (see key resources table) and label filters with a pencil:
    i. *Trichodesmium* on 5.0 μm filters (47 mm diameter, see key resources table).
      **CRITICAL:** Make sure to filter gently, if possible allow gravity filtration. If filtration rate cannot be set to a low speed (vacuum underpressure < 100 mBar), do not connect the tubing to the pump but hold it close to the connection to manually adjust the vacuum. This will help preventing/minimizing the breaking of *Trichodesmium* filaments during this critical step (see troubleshooting problem 1).
    ii. *Synechocystis* and *Cyanothece* on 0.4 μm filters (47 mm diameter, see key resources table).
      **CRITICAL:** We strongly recommend the use of unstained (white) polycarbonate (PC) filters, which retain cells on their surface and help avoid situations where the stain is washed out from the filter, hampering the reactions.
      ***Note:*** For our experiments, we collected between 5 and 8 mL of culture, 3 to 4 h after the start of the light phase. Our laboratory cultures were grown in medium YBCII^32^ (*Trichodesmium* and *Cyanothece*) or BG11^33^ (*Synechocystis*), at 26°C under 150 μmol photons m^−2^ s^−1^, with a 12:12 h light:dark regime and continuous agitation at 100 rpm on a back-forth plate shaker. Cultures cell densities were approximately: 1.8 × 10^5^ cells mL^−1^ for *T. erythraeum* IMS101, 3 × 10^5^ cells mL^−1^ for *T. erythraeum* NIBB1067, 1 × 10^7^ cells mL^−1^ for *Synechocystis*, and 5 × 10^6^ cells mL^−1^ for *Cyanothece*. We estimated *Trichodesmium* cell counts using a Sedgewick Rafter counting chamber, whereas we obtained *Synechocystis* and *Cyanothece* counts by means of an automatic cell counter (see key resources table). At these cell densities, the sampled volumes resulted in suitable cell density on the filters, avoiding overlapping cells and allowing for capturing a large number of cells per field of view during microscopy.

**Figure 2:**
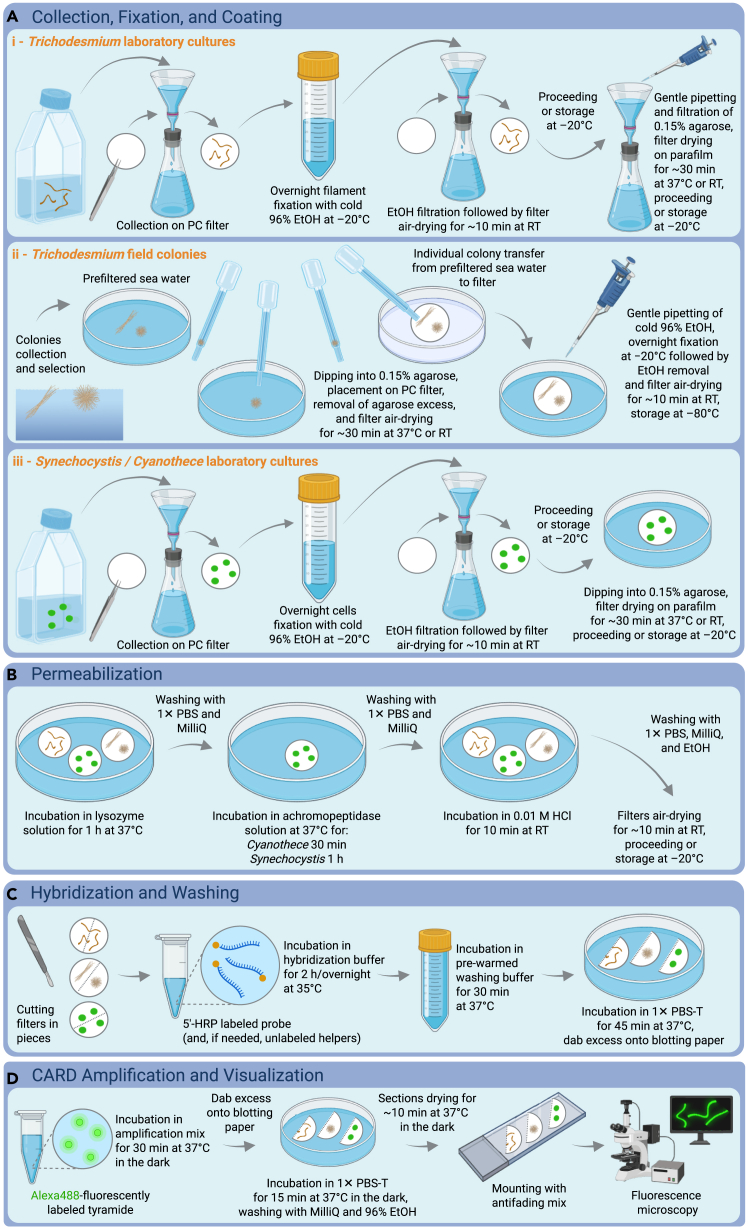
Schematic illustration of the mRNA CARD-FISH protocol steps applied to cyanobacteria PC = polycarbonate filters; EtOH = ethanol; RT = room temperature; 5′-HRP = 5′-horseradish peroxidase. (A) Collection, fixation, and coating. (B) Permeabilization. (C) Hybridization and washing. (D) CARD amplification and visualization.
2. Sample collection of *Trichodesmium* field colonies.
  ***Note:*** Sample collection of *Trichodesmium* field colonies is schematically illustrated in Figure 2Aii.
  ***Note:*** The *Trichodesmium* field colonies employed in this study were collected from the Gulf of Aqaba in the Red Sea (29.56°N, 34.95°E) during 2019 and 2021 fall blooms, and 2022 spring bloom. For details regarding colony collection, we address the reader to Wang et al.^34^ and Zhang et al.^35^
  Briefly:
  a. Collect *Trichodesmium* colonies with a suitable approach for the specific location, such as a 100 μm phytoplankton net suspended from a pier or towed by a motorboat.
  b. Dilute the net concentrate into buckets containing fresh sea water and, once in the lab, hand-pick (with sterile plastic droppers) and select integral and well-shaped colonies. Transfer them into Petri dishes filled with 0.2-μm-filtered seawater.
    ***Note:*** Depending on the purpose of the study, at this point, researchers may perform their desired incubation experiments before proceeding with the protocol.

### Fixation and coating of laboratory cultures and field colonies

**Timing: Overnight**

Here, we describe steps for the sample fixation and coating of *Trichodesmium*, *Synechocystis*, and *Cyanothece* laboratory cultures and *Trichodesmium* field colonies.

3. Fixation and coating of *Trichodesmium*, *Synechocystis*, and *Cyanothece* laboratory cultures.
  ***Note:*** Fixation and coating of *Trichodesmium*, *Synechocystis*, and *Cyanothece* laboratory cultures are schematically illustrated in Figure 2Ai and iii.
  a. Transfer the filters to a 50 mL Falcon tube filled with 25 mL of precooled (−20°C) 96% EtOH.
  b. Incubate overnight at −20°C.
  c. Place back the filters on the filtration setup and filter the 25 mL EtOH.
  d. Air-dry the filters for ∼10 min at RT.
    **Pause point:** Proceed with next step, or store the filters at −20°C.
  e. Coat the filters with warm 0.15% agarose (30°C–40°C):
    i. For *Trichodesmium* culture: place back the filters (cells-up) on the filtration setup, gently pipette the warm agarose (approximately 5 mL) onto the filters, and, by gentle filtration, allow the warm agarose to form a thin film on the filter encoating the filaments while ensuring that the filaments are evenly distributed.
      **CRITICAL:** Since *Trichodesmium* is a large organism, performing the coating on the filtration setup helps prevent/minimize filament loss (see troubleshooting problem 1). This refinement is critical, as the conventional practice of dipping filters into melted agarose resulted in significant loss of filaments. Although much of the agarose may pass through the filter, in our experience, this approach was the most effective way to immobilize the majority of *Trichodesmium* filaments while also preventing the formation of a thick agarose layer, inconvenient during visualization by microscopy.
      ***Note:*** A specific recommendation for this step is to use agarose when it is hand-warm (∼40°C), to prevent filament breakage due to heat as well as agarose solidification during filtration.
    ii. For *Synechocystis* and *Cyanothece* cultures: coat filters by directly immersing them into a Petri dish filled with warm agarose (approximately 10 mL).
      ***Note:*** We did not notice cell breakage or loss during our experiments on *Synechocystis* and *Cyanothece*, hence, specific care does not seem to be required for these single-celled species during filtration and coating.
      ***Note:*** Throughout our protocol, we imply the use of 6 cm diameter Petri dishes.
  f. Place filters (cells-up) on parafilm, and dry them for ∼30 min at 37°C, or at RT.
    **Pause point:** Proceed with next step, or store filters at −20°C.
4. Coating and fixation of *Trichodesmium* field colonies.
  ***Note:*** Coating and fixation of *Trichodesmium* field colonies are schematically illustrated in Figure 2Aii.
  a. Using the plastic dropper, individually pick colonies (in the least possible amount of 0.2-μm-filtered seawater) and dip them into a Petri dish filled with warm 0.15% agarose (30°C–40°C).
  b. Individually aspirate back the colonies (in the least possible amount of agarose) and transfer colonies on a filter (3 to 5 colonies per filter) placed into a Petri dish. Carefully remove the excess of agarose around the colonies.
  c. Dry filters for ∼30 min at 37°C, or at RT, and label them with a pencil.
    **CRITICAL:** Performing coating before fixation helps prevent/minimize colony loss during the next steps of the protocol (see troubleshooting problem 1).
  d. Depending on filter diameter, perform fixation in Petri dishes or 2 mL Eppendorf tubes by adding precooled (−20°C) 96% EtOH.
    **CRITICAL:** Filters should be carefully folded so their back touches the tube wall, and the colonies face the inside of the tube. Filters must not be forced into the tube and crumpled.
    ***Note:*** 25 mm diameter filters (see key resources table) are useful especially when collecting *Trichodesmium* colonies during an oceanographic cruise, since they fit into 2 mL Eppendorf tubes that allow the use of a small volume of EtOH during fixation and easier subsequent storage.
  e. Incubate overnight at −20°C.
  f. Remove EtOH and air-dry filters for ∼10 min at RT.
    **Pause point:** Proceed with next step, or store filters at −80°C.
    ***Note:*** Storage at −80°C is recommended, as field colony collection is typically limited to bloom seasons and extended time periods may pass between their collection and further processing.

### Permeabilization of laboratory cultures and field colonies

**Timing: 1–2 h**

**Timing: 1 h (for step 5)**

**Timing: 30 min–1 h (for step 6)**

**Timing: 10 min (for step 7)**

Here, we describe steps for the sample permeabilization of *Trichodesmium*, *Synechocystis*, and *Cyanothece* laboratory cultures and *Trichodesmium* field colonies.

5. Lysozyme digestion.
  ***Note:*** Permeabilization in lysozyme is schematically illustrated in Figure 2B.
  a. Prepare fresh lysozyme solution in a Falcon tube.

**Table:** 

Reagent	Amount
EDTA (0.5 M, pH 8)	1 mL
TRIS-HCl (1 M, pH 8)	1 mL
Lysozyme (10 mg/mL)	100 mg
MilliQ water	8 mL

    ***Note:*** Store lysozyme powder at −20°C (see key resources table) for up to a year.
  b. Mix and pour the lysozyme solution into a Petri dish.
  c. Immerse the filters in the lysozyme solution and close the Petri dish to prevent evaporation during incubation.
  d. Incubate the samples for 1 h at 37°C.
  e. Successively wash samples by transferring the filters into a Petri dish containing 1 × PBS, and then into a Petri dish containing MilliQ water.
6. Additional achromopeptidase digestion of *Synechocystis* and *Cyanothece*.
  ***Note:*** The additional incubation in achromopeptidase is schematically illustrated in Figure 2B.
  a. Prepare fresh achromopeptidase solution in a Petri dish.

**Table:** 

Reagent	Amount
Achromopeptidase-stock (30 KU)	20 μL
NaCl-TRIS buffer	10 mL
  b. Immerse the filters in the achromopeptidase solution and close the Petri dish.
  c. Incubate at 37°C:
    i. *Cyanothece* samples for 30 min.
    ii. *Synechocystis* samples for 1 h.
  d. Successively wash samples by transferring the filters into a Petri dish containing 1 × PBS, and then into a Petri dish containing MilliQ water.
7. Incubation of all samples in HCl to deactivate endogenous peroxidases.
  ***Note:*** The incubation in HCl is schematically illustrated in Figure 2B.
  a. Prepare 0.01 M HCl in a Petri dish.

**Table:** 

Reagent	Amount
HCl (1 M)	100 μL
MilliQ water	9.9 mL

    ***Note:*** Keep few mL of HCl 1 M at RT (for up to several months; see key resources table) for quick preparation of the 0.01 M HCl solution.
    ***Note:*** Re-start from this point in case of double hybridization.
  b. Incubate filters in 0.01 M HCl for 10 min at RT.
  c. Successively wash samples by transferring the filters into a Petri dish containing 1 × PBS, a Petri dish containing MilliQ water, and then into a Petri dish containing EtOH (70% EtOH commonly used in the laboratory is acceptable).
  d. Air-dry the samples for ∼10 min at RT.
    **Pause point:** Proceed with next step, or store filters at −20°C.

### Probe hybridization and washing

**Timing: 3.5 h–overnight (∼15 h)**

Here, we describe steps for probe hybridization and washing.

***Note:*** Sample hybridization and washing are schematically illustrated in Figure 2C.

***Note:*** Note that we do not expect that the hybridization condition we defined here for the *rbcL* probe to hold for other mRNAs. Instead, hybridization conditions should be initially tested for each new probe and transcript.

8. Cut filters into pieces and label them with a pencil.
9. Prepare probe mix in a 0.5 mL tube.

**Table:** 

Reagent	Amount
Hybridization buffer (% HB)	300 μL
HRP-labeled probe	X μL
Helpers (if needed)	X μL

***Note:*** The amount of probes and helpers depend on the desired final concentration, which in this study was 0.06 pmol/μL and corresponded to 2–3 μL. Do not add any probe for the No-probe negative control.

***Note:*** The % of formamide in the hybridization buffer (% HB) may vary depending on the probe. Here, for EUB and NON-EUB probes, formamide represents 55% of the % HB.^24^^,^^25^ For the *rbcL* probe designed for the purpose of this study, the % was defined after testing (Figures S1 and S2), and was set to 55%. The same % HB was also used for the negative controls NON-*rbcL* and No-probe.

10. Vortex briefly.
11. Place filter pieces into the tubes.

**CRITICAL:** To avoid potential damage, especially for *Trichodesmium* filaments and colonies, we recommend to place only one filter piece per tube, and to carefully slide the piece onto the tube walls (do not fold/crumple the filter piece! See troubleshooting problem 1).

12. Incubate at 35°C under continuous rotation for:
  a. EUB and NON-EUB samples, 2 h.^24^^,^^25^
  b. *rbcL*, NON-*rbcL*, and No-probe samples, overnight (timing defined in the present study after testing, Figures S1 and S2).
13. Prepare fresh washing buffer and preheat at 37°C during the hybridization incubation.

**Table:** 

Reagent	Amount
EDTA (0.5 M, pH 8)	500 μL
TRIS-HCl (1 M, pH 7.4)	1 mL
NaCl (5 M)	X μL
MilliQ water	to 50 mL
10% SDS	50 μL

***Note:*** SDS should be added at the end. Adding it to the concentrated salt and buffers mixture may cause its precipitation.

***Note:*** The amount of NaCl (5 M) depends on hybridization buffer (% HB), add according to the table below.^29^

**Table:** 

% HB	μL of NaCl (5 M)
20	1350
25	950
30	640
35	420
40	270
45	160
50	90
55	30
60	0
65	0
70	0

14. After hybridization, transfer the filter pieces into the washing buffer for 30 min at 37°C.
15. Incubate the filter pieces in a covered Petri dish in 0.01% PBS-T (approximately 10 mL) for 45 min at 37°C.

### CARD amplification and visualization

**Timing: 1 h (microscope time not accounted)**

Here, we describe steps for CARD amplification and visualization.

***Note:*** CARD amplification and visualization are schematically illustrated in Figure 2D.

16. Prepare amplification mix a few minutes before the end of the incubation in 0.01% PBS-T.

**Table:** 

Reagent	Amount
Amplification buffer	1 mL
0.15% H_2_O_2_	10 μL
Fluorochrome Alexa488	1 μL

17. Vortex briefly.
18. Dab the excess of 0.01% PBS-T remaining on filter pieces onto white blotting paper.

**CRITICAL:** To prevent/minimize sample damage (especially, for *Trichodesmium* filaments/colonies), dab on the filter pieces edge, do not flip (see troubleshooting problem 1)!

***Note:*** In case of a high number of filter pieces, scale up the amounts and perform the amplification incubation in a covered Petri dish.

***Note:*** In case of double hybridization, a second fluorochrome of different excitation/emission band must be used.

19. Incubate filter pieces for 30 min at 37°C in the dark.
20. Dab the excess of amplification mix on filter pieces onto white blotting paper.

**CRITICAL:** Dab on the filter pieces edge, do not flip (see troubleshooting problem 1)!

21. Incubate filter pieces in a covered Petri dish containing 0.01% PBS-T (approximately 10 mL) for 15 min at 37°C in the dark.
22. Successively wash filter pieces by transferring them into a Petri dish containing MilliQ water, and then into a Petri dish containing EtOH (70% EtOH commonly used in the laboratory is acceptable).
23. Dry filter pieces in the dark for approximately for ∼10 min at 37°C.
24. Store filter pieces at −20°C, or place them on glass slides (see key resources table) and embed them in antifading mounting mix. Carefully apply cover slip.

***Note:*** Use regular cover slips in case of visualization by epifluorescence microscopy, while high precision cover slips are more suitable in case of visualization by confocal microscopy (see key resources table).

**CRITICAL:** Avoid the formation of air bubbles while applying the cover slip by pipetting a large drop of mounting mix onto the filter pieces. After placing the cover slip on the filter pieces, allow the mounting mix to spread by capillary forces. Do not apply pressure to the cover slip, as this might damage *Trichodesmium* filaments and colonies (see troubleshooting problem 1).

25. Visualize samples using an epifluorescence or confocal microscope.

***Note:*** For the purpose of this study, both epifluorescence and confocal microscopes were employed. Concerning epifluorescence microscopy, *Trichodesmium* images were obtained at 40× magnification, in automatic exposure time mode, using the following settings (excitation/emission): for Alexa488 stained cells 488/515-long pass nm; for 546-autofluorescence 546/570–640 nm. Once acquired, images were not modified, except for the equally applied adjustment of brightness levels. As concerns confocal microscopy, images were obtained at 40× (for *Trichodesmium*) and at 100× (for *Synechocystis* and *Cyanothece*) magnification, in speed 3, using the following settings (excitation/emission): Alexa488 stained cells 488/508–543 nm; 543-autofluorescence 543/552–615 nm. Once acquired, images were individually adjusted for brightness to better highlight non-hybridized cells. In contrast, no post-acquisition modifications were applied to the lambda scan analyses. Images were acquired and processed using ZEN.blue and Zen.black (see key resources table) software for epifluorescence and confocal microscopy, respectively.

***Note:*** Because we acquired images in automatic exposure mode, we could not perform image analysis to subtract autofluorescence from the hybridization channel. Therefore, we based our analysis on visual inspections (presence/absence) of merged images. To avoid similar issues, particularly for target mRNAs, make sure to use identical acquisition settings to ensure comparability of images for future analysis. Although subtracting autofluorescence would be more correct, we also remind the reader that CARD-FISH is based on an amplified signal and, unlike FISH, interpreting higher or lower fluorescent intensity levels as higher or lower mRNA expression levels would be inappropriate (see limitations).

26. After visualization, store slides at −20°C up to 4–5 years.

## Expected outcomes

For the purpose of this study, we performed a large number of tests in order to find optimal protocol conditions. For *Trichodesmium* (laboratory strains *T. erythraeum* IMS101 and NIBB1067, and field-collected colonies), we evaluated a range of fixation and enzymatic permeabilization procedures. However, almost none of these alternatives resulted in successful hybridization (Figures S3–S11), with the only exception being the test shown in Figure S3B (i.e., 96% EtOH 1 h + Lysozyme 1 h + Achromopeptidase 30 min) performed on *T. erythraeum* IMS101. Yet, since it was not possible to reproduce the same results with the *T. erythraeum* NIBB1067 strain (Figure S3D), this specific combination was not further considered nor repeated. An important insight from these extensive tests is that, comparing lysozyme and achromopeptidase, only the latter led to improved cell digestion (Figures S5C, S7F, and S9D). Although in the final protocol achromopeptidase was not considered necessary for *Trichodesmium*, this finding guided the design of our subsequent tests on *Synechocystis* and *Cyanothece*, in which we performed different incubation times with achromopeptidase (Figure S11). Therefore, when applying our protocol to other cyanobacterial species, we recommend leaving the lysozyme digestion step unchanged and, if necessary, to start by adjusting the achromopeptidase digestion step.

**Figure 3:**
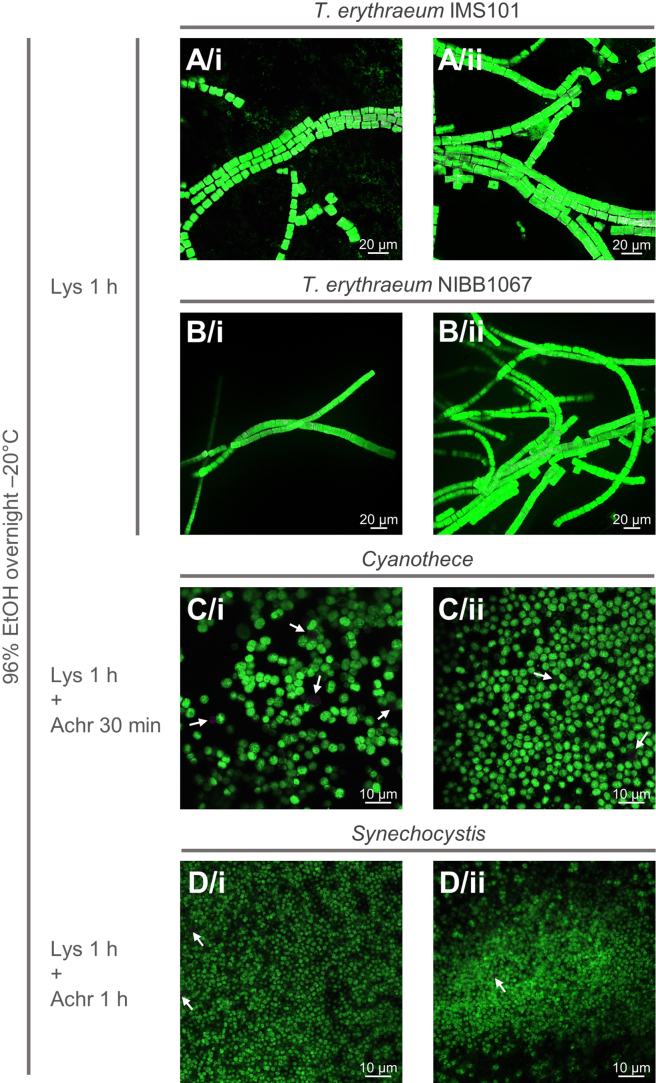
Microscopy images of *T. erythraeum* (IMS101 and NIBB1067 strains), *Cyanothece*, and *Synechocystis* laboratory cultures after fixation and digestion optimized conditions Images are shown as a merge of EUB signal (Alexa488, green) and autofluorescence (543, pink). White arrows indicate non-labeled cells. Each row represents two images from the same sample. Scale bars: 20 μm in A and B; 10 μm in C and D. Lys = lysozyme; Achr = achromopeptidase. (A) *T. erythraeum* IMS101. (B) *T. erythraeum* NIBB1067. (C) *Cyanothece*. (D) *Synechocystis*.

**Figure 4:**
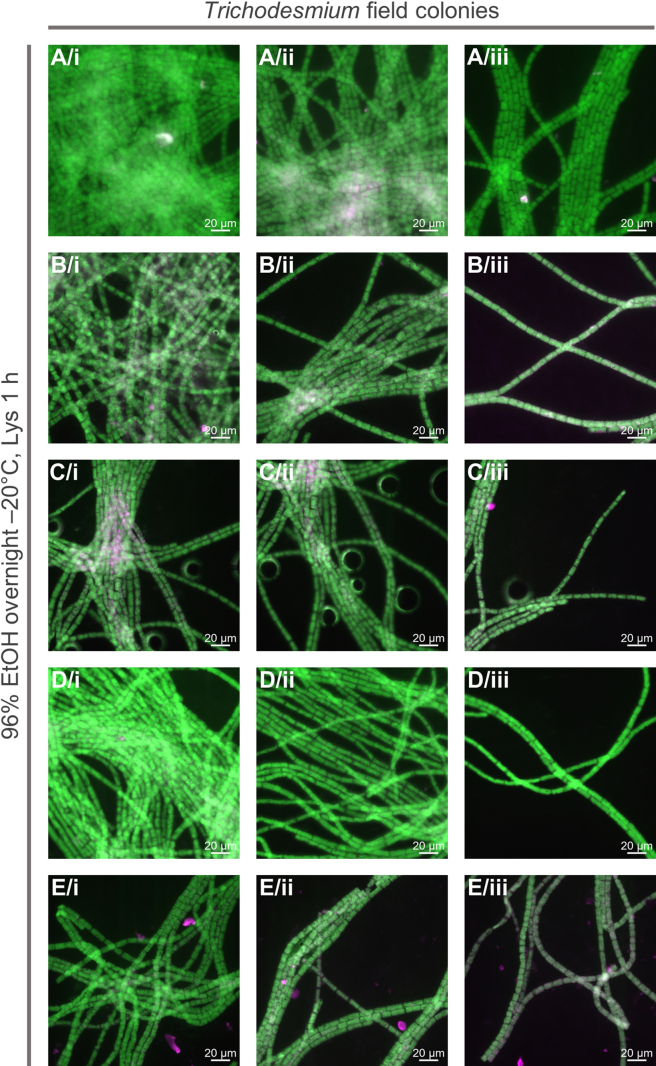
Microscopy images of *Trichodesmium* field colonies after fixation and digestion optimized conditions Images are shown as a merge of EUB signal (Alexa488, green) and autofluorescence (546, pink). All scale bars: 20 μm. EtOH = ethanol; Lys = lysozyme. (A–E) Each row represents different regions of the same colony; each colony was analyzed during different experiments.

Based on all performed tests, we defined the optimal combination of fixation and digestion conditions as described in the step-by-step method details: overnight incubation in 96% EtOH at −20°C and 1 h incubation in lysozyme for all tested cyanobacteria, followed by 30 min and 1 h incubation in achromopeptidase for *Cyanothece* and *Synechocystis*, respectively (Figures 3, 4, S4A, and S6A). Although these digestion steps represent the best conditions we could define for *Synechocystis* and *Cyanothece*, a small fraction of cells might still not be labeled (Figures 3C and 3D). In our EUB-probed experiments, 0.7% and 0.9% of *Synechocystis* and *Cyanothece* cells respectively were not labeled (*Synechocystis*: analyzed pictures = 2, total cells = 2456, non-labeled cells = 4; *Cyanothece*: analyzed pictures 3, total cells = 1130, non-labeled cells = 10; counts performed using automatized particle analysis in ImageJ, see key resources table, with manual addition of uncounted particles observed during visual inspection). Since longer incubations with achromopeptidase resulted in overdigested cells (Figures S11G and S11H), we recommend subtracting the percentage of non-labeled cells in the positive control (EUB-probed) from the experimental results targeting a gene of interest (see troubleshooting problem 2). Although the main steps of our protocol do not drastically differ from other protocols generally used in the field, our extensive testing highlighted that: (1) fixation is crucial for successful permeabilization (and/or hybridization); (2) optimal permeabilization conditions may differ between different cyanobacterial species; and (3) specific handling refinements are required for large cyanobacteria such as *Trichodesmium* (see troubleshooting problem 1). To our knowledge, such extensive testing is unprecedentedly reported for cyanobacteria. Altogether, the three refinements we identified are essential not only to ensure reliable mRNA CARD-FISH results for cyanobacteria, but also relevant to classical rRNA CARD-FISH for quantification of unicellular cyanobacteria in environmental samples.

**Figure 5:**
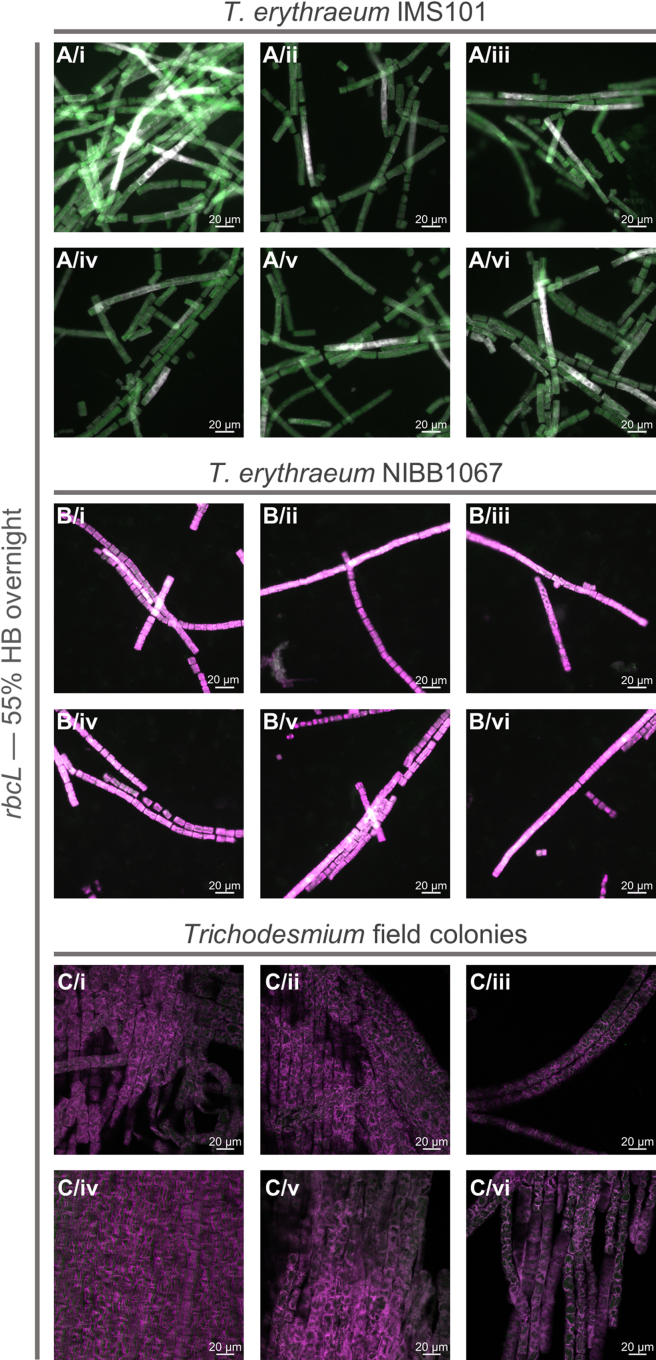
*rbcL* gene expression pattern visualized on *Trichodesmium* laboratory cultures (*T. erythraeum* IMS101 and NIBB1067 strains) and field colonies Images are shown as a merge of *rbcL* signal (Alexa488, green) and autofluorescence (546 or 543 for epifluorescence and confocal microscopy, respectively, pink). Images of laboratory strains represent different filaments from the same sample, while each row of field colony images shows different regions of the same colony (analyzed during the same experiment). All scale bars: 20 μm. % HB = % of formamide in hybridization buffer. (A) *T. erythraeum* IMS101. (B) *T. erythraeum* NIBB1067. (C) *Trichodesmium* field colonies.

**Figure 6:**
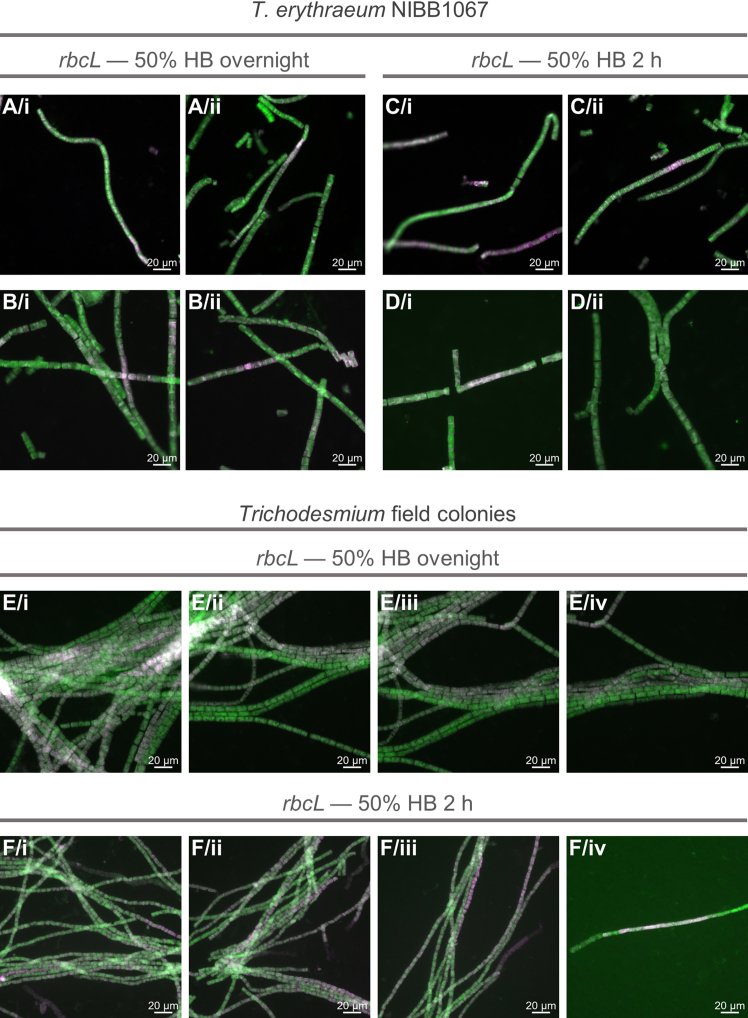
*rbcL* gene expression pattern visualized on *T. erythraeum* NIBB1067 strain and field colonies Images are shown as a merge of *rbcL* signal (Alexa488, green) and autofluorescence (546, pink). Each row in NIBB1067 strain represents filaments from the same sample, hence, in total we show images from two different experiments. Each row of field colony images shows different regions of the same colony. All scale bars: 20 μm. % HB = % of formamide in hybridization buffer. (A and B) *T. erythraeum* NIBB1067, *rbcL*, overnight, 50% HB. (C and D) *T. erythraeum* NIBB1067, *rbcL*, 2 h, 50% HB. (E) *Trichodesmium* field colony, *rbcL*, overnight, 50% HB. (F) *Trichodesmium* field colony, *rbcL*, 2 h, 50% HB.

**Figure 7:**
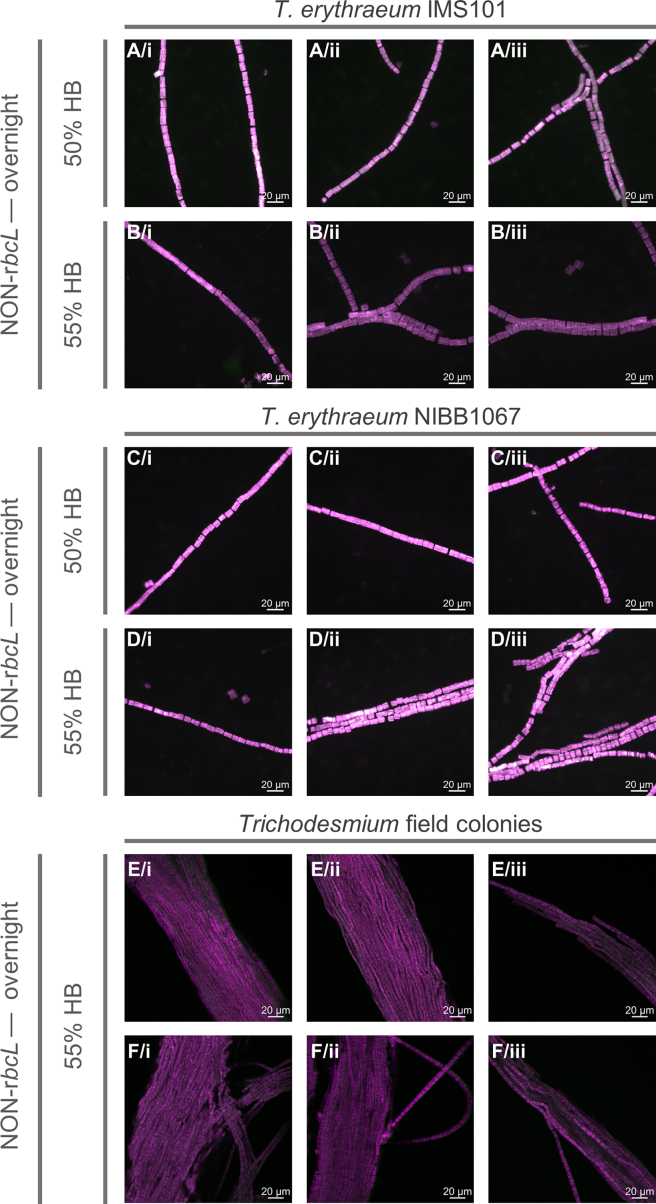
NON-*rbcL* negative control tested on *Trichodesmium* laboratory cultures (*T. erythraeum* IMS101 and NIBB1067 strains) and field colonies Images are shown as a merge of NON-*rbcL* signal (Alexa488, green) and autofluorescence (546 or 543 for epifluorescence and confocal microscopy, respectively, pink). Images of laboratory strains represent different filaments from the same sample, while each row of field colony images shows different regions of the same colony (analyzed during the same experiment). All scale bars: 20 μm. % HB = % of formamide in hybridization buffer. (A) *T. erythraeum* IMS101, NON-*rbcL*, overnight, 50% HB. (B) *T. erythraeum* IMS101, NON-*rbcL*, overnight, 55% HB. (C) *T. erythraeum* NIBB1067, NON-*rbcL*, overnight, 50% HB. (D) *T. erythraeum* NIBB1067, NON-*rbcL*, overnight, 55% HB. (E and F) *Trichodesmium* field colonies, NON-*rbcL*, overnight, 55% HB.

**Figure 8:**
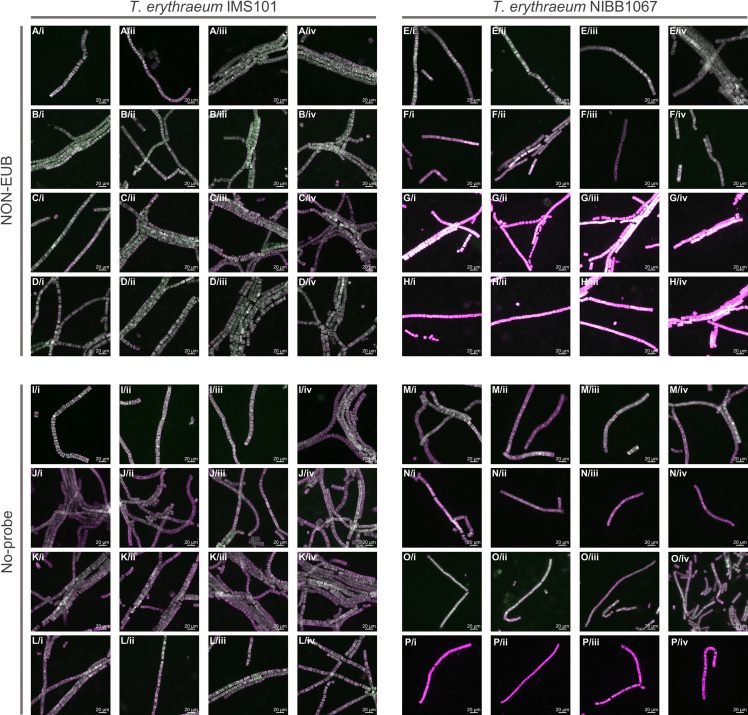
NON-EUB and No-probe negative controls tested on *T. erythraeum* IMS101 and NIBB1067 strains Images are shown as a merge of both autofluorescence signals (Alexa488, green; 546, pink). Each row represents filaments from the same sample, hence, in total we show images from four different experiments. All scale bars: 20 μm. (A–D) *T. erythraeum* IMS101, NON-EUB. (E–H) *T. erythraeum* NIBB1067, NON-EUB. (I–L) *T. erythraeum* IMS101, No-probe. (M–P) *T. erythraeum* NIBB1067, No-probe.

**Figure 9:**
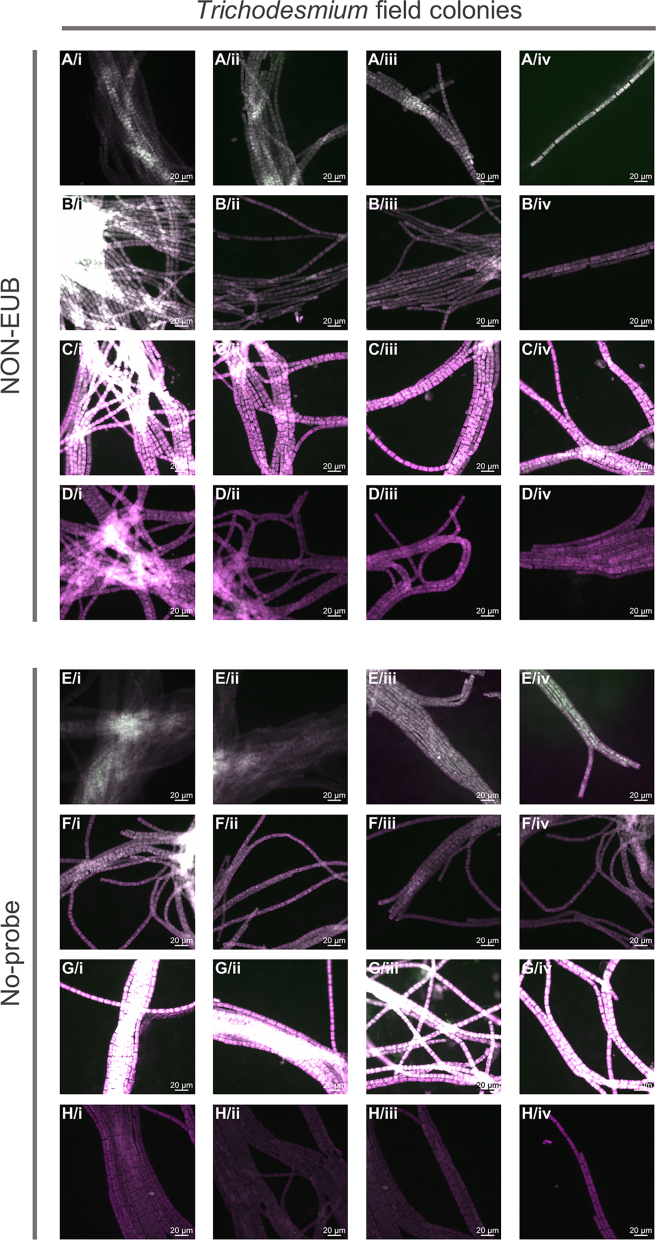
NON-EUB and No-probe negative controls tested on *Trichodesmium* field colonies Images are shown as a merge of both autofluorescence signals (Alexa488, green; 546, pink). Each row represents different regions of the same colony, hence, in total we show images from four different experiments. All scale bars: 20 μm. (A–D) NON-EUB. (E–H) No-probe.

**Figure 10:**
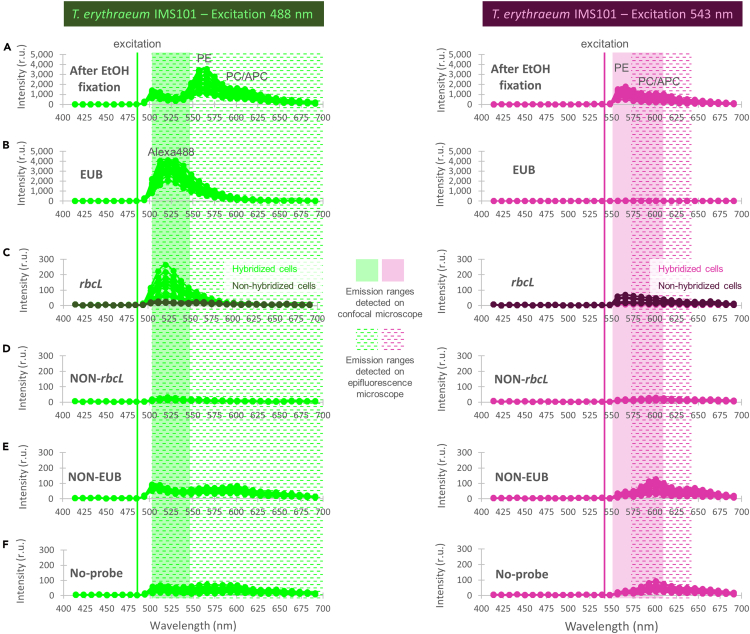
Emission spectra of *T. erythraeum* IMS101 after excitation at 488 and 543 nm (left and right columns, respectively) Each graph displays measurements from ten cells (five hybridized and five non-hybridized from the *rbcL* experiment) per filament, across three filaments (30 cells in total). Note that y-axes of panels A and B differ from the below panels (C–F). APC = allophycocyanin; PC = phycocyanin; PE = phycoerythrin; EtOH = ethanol; r.u. = relative unit. (A) After EtOH fixation. (B) EUB. (C) *RbcL*. (D) NON-*rbcL*. (E) NON-EUB. (F) No-probe.

After optimization using the EUB probe, we applied the protocol to investigate *rbcL* gene expression patterns in *Trichodesmium* as an example of mRNA transcriptional detection. Our analysis revealed heterogeneous *rbcL* gene expression in *T. erythraeum* IMS101 strain, with cells located in the middle of filaments showing no expression (Figure 5A). Interestingly, protein immunolabeling studies suggested that cells in the center of filament in *T. erythraeum* IMS101 fix N_2_, as nitrogenase–the enzyme responsible for N_2_ fixation–was localized exclusively in these central regions.^36^^,^^37^^,^^38^^,^^39^ The absence of *rbcL* expression in these central cells suggests a spatial decoupling between photosynthesis and N_2_ fixation, and thus a potential mechanism for protecting nitrogenase from O_2_. Yet, the expression pattern observed in this study appears to contrast with RubisCO protein immunolocalization, which was found to be uniformly distributed along the filaments.^40^^,^^41^ No *rbcL* signal was instead detected during our investigation in *T. erythraeum* NIBB1067 strain or in field colonies under the same hybridization conditions (Figures 5B and 5C, see limitations and troubleshooting problem 3). However, we were able to detect *rbcL* signal under slightly different hybridization conditions, in which both *T. erythraeum* NIBB1067 strain and field colonies also displayed heterogeneous *rbcL* expression (Figure 6). Although the two hybridization conditions are similar, one possible explanation regarding the difference in optimal % HB is that, since our probe sequence is degenerated, it is not a single sequence but rather a mixture of slightly different oligos, so the optimal hybridization conditions may vary depending on which variants match a given strain best. Importantly, none of the NON-*rbcL*, NON-EUB, and No-probe negative controls exhibited hybridization signal (Figures 7, 8, 9, and S12). The reliability of our results was further supported by emission spectra analysis performed on the *T. erythraeum* IMS101 strain, which confirmed the emission peak of Alexa488 in both EUB-probed positive control and *rbcL*-probed cells (Figure 10). As expected, the signal intensity in *rbcL*-probed cells was lower, likely reflecting the lower abundance of mRNA compared with the 16S rRNA in ribosomes that is targeted by the EUB probe. No corresponding peak was detected in the cells not hybridized with the *rbcL* probe, or in the negative controls NON-EUB and No-probe (Figure 10). A very weak peak was detected in the NON-*rbcL* negative condition (Figure 10D). We suggest that weak peaks from NON-probes can be used to set a threshold for what it is considered a positive mRNA signal. A last noteworthy observation is the reduced pigment peak intensities (PE, APC/PC, Figure 10) in samples after CARD-FISH compared with those analyzed after fixation alone, a result that correlates well with those of the autofluorescence reduction tests (Figure 1).

In summary, we believe our optimized mRNA CARD-FISH protocol can provide new insights into the regulation of physiological processes by visualizing their gene expression patterns at the single-cell level. By applying this protocol, we were for instance able to shed light on the orchestration between N_2_ fixation and photosynthesis in *T. erythraeum* IMS101 by visualizing *nifH* (encoding Fe protein of nitrogenase) and *psbA* (encoding D1 protein of PSII) genes by both single and double CARD-FISH (Lopez-Adams et al. in review). In addition, the handling tips we provide for working with *Trichodesmium* colonies proved particularly useful when applying CARD-FISH for regular rRNA targeting use, i.e., we investigated bacteria associated with natural *Trichodesmium* colonies (Zhang et al. in prep.). Here, we offer our optimized protocol to researchers working with various aspects of the cellular physiology and/or functional ecology of marine and freshwater cyanobacteria. Considering the growing interest in the phenomenon of cell-to-cell heterogeneity, mRNA CARD-FISH provides a powerful means to visualize differences between cells within the same population, which are undetectable by traditional bulk gene expression analysis.

## Limitations

A general limitation of the CARD-FISH approach is that due to the signal amplification step, and unlike FISH, CARD-FISH can only be qualitative (presence/absence). Thus, interpreting higher or lower CARD-FISH signal intensities as differences in expression levels (across cell populations, species, or experimental conditions) is not appropriate.

Another major limitation related to the method is the difficulty in determining whether non-detectable mRNA signal is due to low transcript abundance or an inefficient probe sequence. To answer this tricky question, we propose some ideas in the next section (see troubleshooting problem 3). We also acknowledge that the fact that our protocol does not employ PFA nor GA (both incompatible with our protocol and/or strains) might contribute to potential mRNA degradation, and therefore in an artefactual loss of hybridization signal. We also cannot rule out the possibility that factors such as the physiological state of the cells, or low transcript stability, may compromise reproducibility of the results.

Finally, another limitation concerns the potential necessity to adapt our protocol for other cyanobacterial species. In such case, adjustments may be required to optimize filtration and coating procedures, as well as enzymatic permeabilization conditions. Table 2 provides a selection of possible conditions that can be tested. In this context, it is worth noting that, when working with *Trichodesmium* field colonies, we observed that dense colonies were not hybridized in their core/center region. This issue, that is likely due to the high filament density which prevents the penetration of chemicals, may also apply to other aggregate-forming cyanobacteria, even under optimal protocol conditions. We therefore recommend either selecting colonies with a low filament density or looser filaments structure (i.e., less tightly packed to each other), or opting for embedding and sectioning of samples.

## Troubleshooting

### Problem 1

*Trichodesmium* filament fragmentation and/or colony loss.

### Potential solution

Generally, we recommend extreme care when working with *Trichodesmium* in order to minimize the potential issues related to filament fragmentation and colony loss. We provide practical tips on sample filtration, coating, and handling refined during the development of our protocol (see critical points of steps 1ai and 1ei for laboratory cultures, critical point of step 4c for field-collected colonies, and critical points of steps 11, 18, 20 and 24 in the step-by-step method details).

### Problem 2

Incomplete labeling of *Synechocystis* and *Cyanothece* cells in EUB-probed positive control, likely results from old cells with thicker cell walls, dead cells (= having no ribosomes) prior to fixation, or weak enzymatic digestion.

### Potential solution

We attempted to overcome this problem by extending the incubation time with achromopeptidase. However, these longer incubations resulted in overdigested cells (Figures S11F–S11H). Hence, we recommend addressing this issue by subtracting the percentage of non-labeled cells in the positive control (EUB-probed) from the experimental results targeting a gene of interest (see expected outcomes).

### Problem 3

Non-detectable mRNA signal.

### Potential solution

If the EUB-probed positive control shows no issues related to cell permeabilization, the absence of a detectable mRNA signal (see limitations) may be due to very low expression levels, which in turn can be explained by sampling outside the target gene’s peak (or maximum) expression window. In such cases, sampling time can be optimized based on the expression dynamics of the target gene by complementing mRNA CARD-FISH with regular qPCR analysis. If, after defining an optimal sampling time, detection is still not achieved, the target gene may be expressed at levels too low to be sufficiently enhanced by CARD-FISH amplification, a limitation that cannot be overcome.

## Resource availability

### Lead contact

Further information and requests for resources and reagents should be directed to and will be fulfilled by the lead contact, Meri Eichner (eichner@alga.cz).

### Technical contact

Technical questions on executing this protocol should be directed to and will be answered by the technical contact, Anxhela Hania (hania@alga.cz).

### Materials availability

In this study we generated probes and helpers, the respective purchase details and oligonucleotide sequences are listed in the key resources table and Table 1.

### Data and code availability

This study did not generate/analyze dataset/code.

## Acknowledgments

We sincerely thank Prof. Yeala Shaked for her support and coordination of the sampling efforts at her laboratory. We are grateful to Antonio Colussi and Futing Zhang for collecting the *Trichodesmium* field colonies in fall 2021. We also thank Coco Koedooder for providing *Trichodesmium*’s *rbcL* gene sequences, which can be found under the NCBI BioProject ID number PRJNA804487.^27^ M.E. was supported by OP VVV (reg. no. CZ.02.2.69/0.0/0.0/20_079/0017812) as well as by P JAC project “Photomachines” (reg. no. CZ.02.01.01/00/22_008/0004624); M.E., A.H., and L.D. were supported by 10.13039/100017097GACR (grant numbers 20-02827Y and 24-11363S). O.P. was supported by 10.13039/100017097GACR (grant number 23-06593S). The graphical abstract and Figure 2 were created in BioRender.com.

## Author contributions

Project conceptualization, supervision, and *Trichodesmium* field colony collection in fall 2019, M.E.; investigation, method development, data analysis, and *Trichodesmium* field colony collection in spring 2022, A.H.; *Synechocystis* and *Cyanothece* experiments, L.D.; conceptual and technical guidance, K.P.; writing – original draft and figure preparation, A.H.; writing – review and editing, A.H., L.D., M.E., K.P., and O.P. All authors have read and agreed to the final version of the manuscript.

## Declaration of interests

The authors declare no competing interests.

## Declaration of generative AI and AI-assisted technologies in the writing process

During the preparation of this work, the author(s) used ChatGPT in order to enhance the clarity and readability of the manuscript. After using this tool, the author(s) reviewed and edited the content as needed, and take(s) full responsibility for the content of the published article.
