# Supplementary material for: Protocol to investigate gene expression heterogeneity in cyanobacteria using mRNA CARD-FISH
Source: STAR Protoc. 2025 Nov 20;6(4):104212. doi: 10.1016/j.xpro.2025.104212 (PMC12681788; doi:10.1016/j.xpro.2025.104212)
Supplement: Document S1. Figures S1–S12 [file mmc1.pdf]

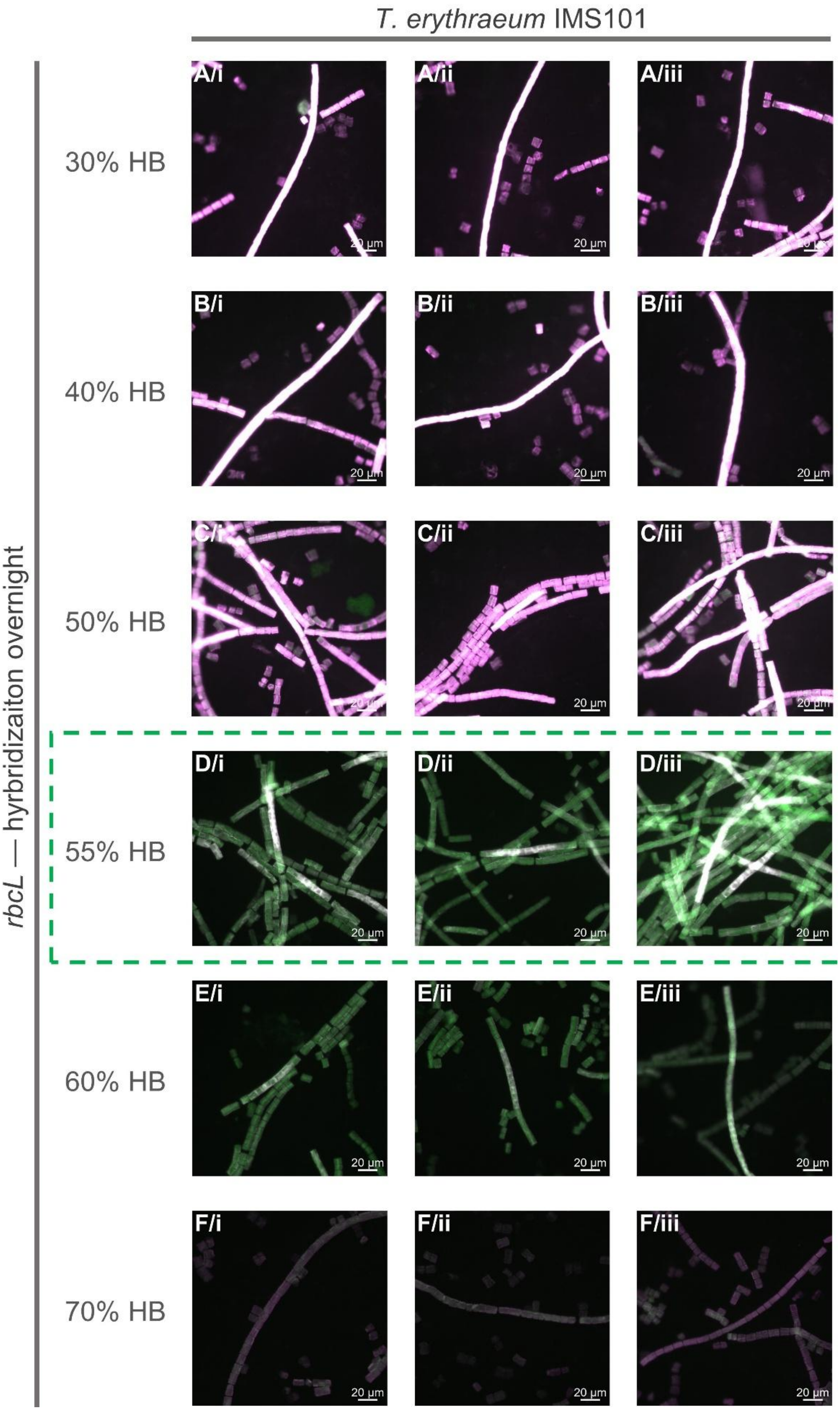

Figure S1. Optimization of hybridization conditions (overnight incubation) for *rbcL* probe performed on *T. erythraeum* IMS101, relates to Step 12b. Images are shown as a merge of *rbcL* signal (Alexa488, green) and *Trichodesmium*'s autofluorescence (546, pink). The green dashed rectangle highlights the optimal hybridization condition, according to our tests. Each row represents filaments from the same sample. All scale bars: 20 µm. % HB = % of formamide in the hybridization buffer. The scale bar in panel R applies to all panels. A) 30% HB. B) 40% HB. C) 50% HB. D) 55% HB. E) 60% HB. F) 70% HB.

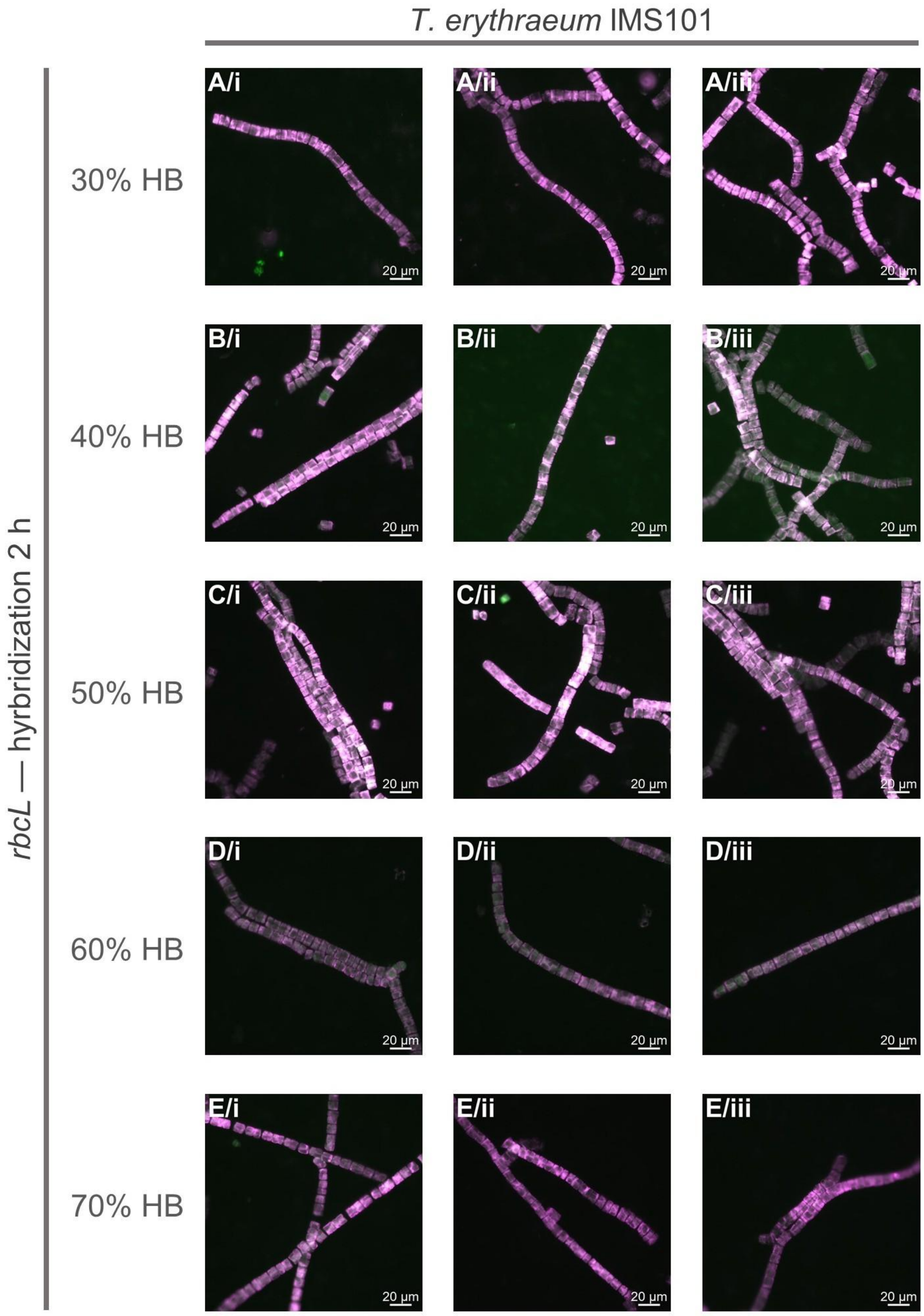

Figure S2. Optimization of hybridization conditions (2 h incubation) for *rbcL* probe performed on *T. erythraeum* IMS101, relates to Step 12b. Images are shown as a merge of *rbcL* signal (Alexa488, green) and *Trichodesmium*'s autofluorescence (546, pink). Each row represents filaments from the same sample. All scale bars: 20 µm. % HB = % of formamide in the hybridization buffer. A) 30% HB. B) 40% HB. C) 50% HB. D) 60% HB. E) 70% HB.

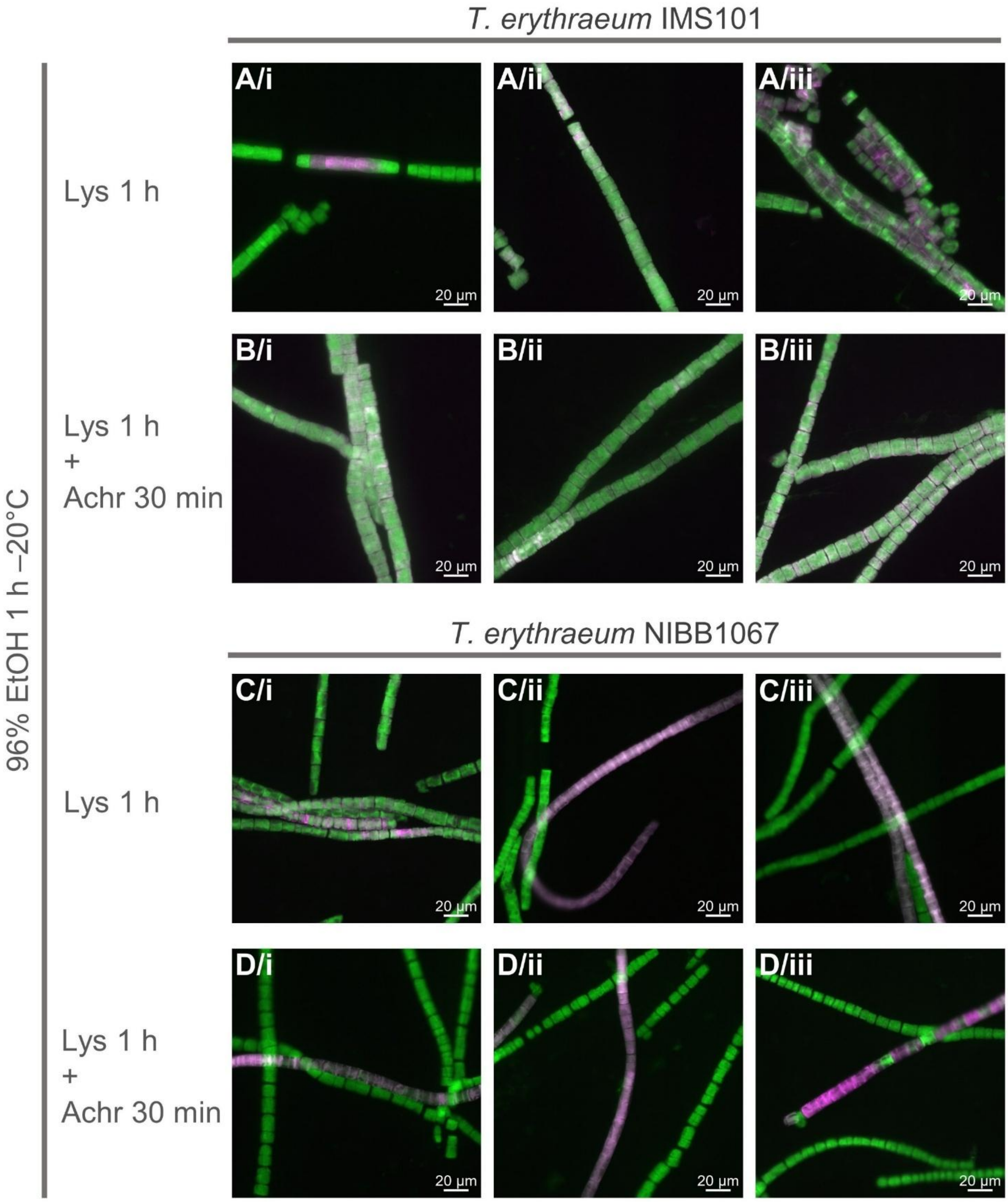

Figure S3. Digestion tests performed on *T. erythraeum* IMS101 and NIBB1067 strains, related to Steps 3, 5, and 6. Interestingly, the 1 h lysozyme incubation followed by 30 min incubation in achromopeptidase (Lys 1 h + Achr 30 min) was successful for the IMS101 strain (B), but not for the NIBB1067 strain (D). Images are shown as a merge of EUB signal (Alexa488, green) and *Trichodesmium*'s autofluorescence (546, pink). Each row represents filaments from the same sample. All scale bars: 20 µm. EtOH = ethanol; Lys = lysozyme; Achr = achromopeptidase. A) *T. erythraeum* IMS101, 96% EtOH 1 h −20°C, Lys 1 h. B) *T. erythraeum* IMS101, 96% EtOH 1 h −20°C, Lys 1 h + Achr 30 min. C) *T. erythraeum* NIBB1067, 96% EtOH 1 h −20°C, Lys 1 h. D) *T. erythraeum* NIBB1067, 96% EtOH 1 h −20°C, Lys 1 h + Achr 30 min.

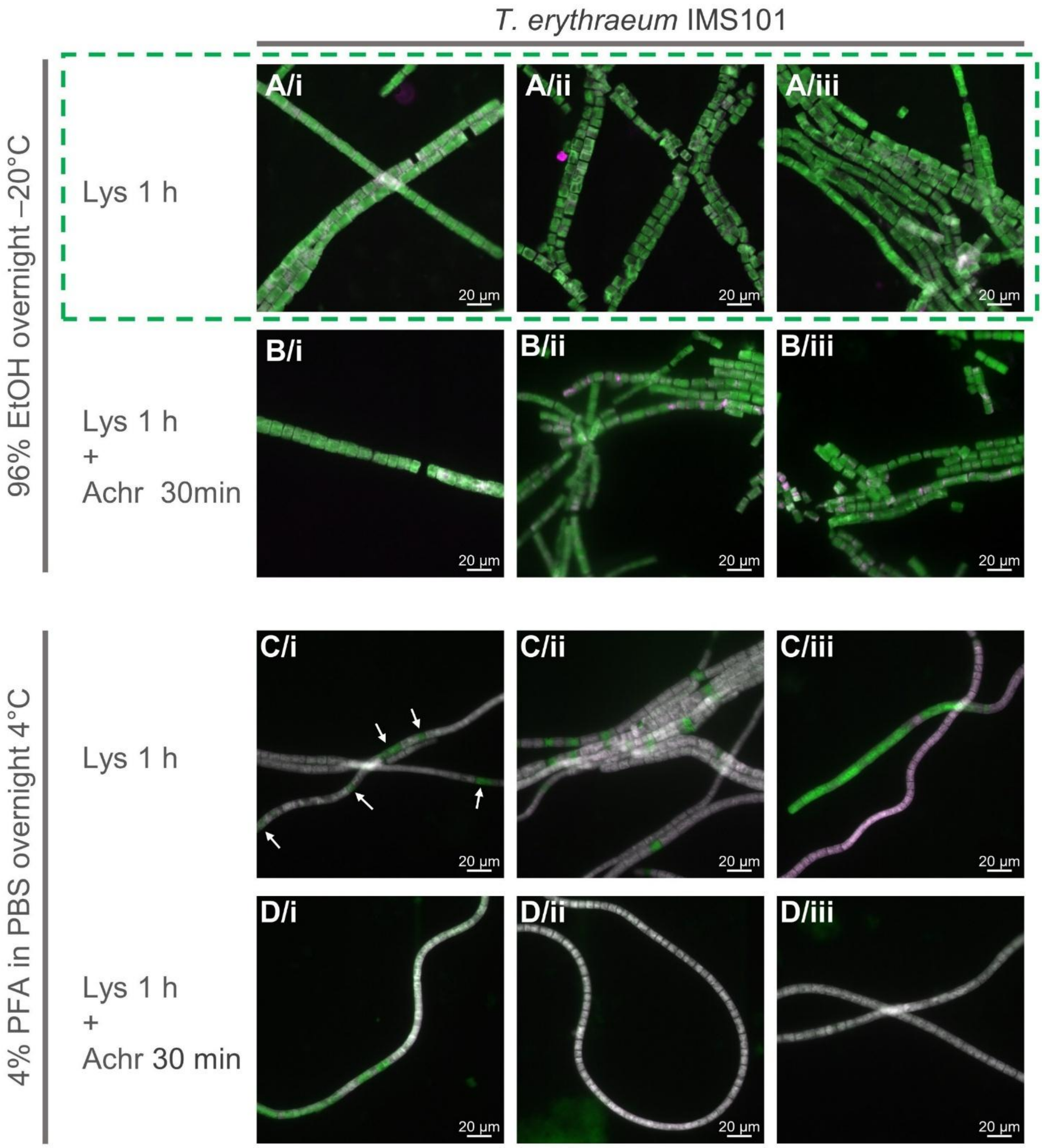

Figure S4. Digestion tests performed on *T. erythraeum* IMS101 strain, related to Steps 3, 5, and 6. Images are shown as a merge of EUB signal (Alexa488, green) and *Trichodesmium*'s autofluorescence (546, pink). The green dashed rectangle highlights the optimal fixation and digestion conditions, according to our tests. The first two rows represent filaments from three different samples of three different experiments, while the others represent filaments from the same sample. White arrows point to hybridized *Trichodesmium* cells. All scale bars: 20 µm. EtOH = ethanol; PFA = paraformaldehyde; Lys = lysozyme; Achr = achromopeptidase. A) 96% EtOH overnight −20°C, Lys 1 h. B) 96% EtOH overnight −20°C, Lys 1 h + Achr 30 min. C) 4% PFA in PBS overnight 4°C, Lys 1 h. D) 4% PFA in PBS overnight 4°C, Lys 1 h + Achr 30 min.

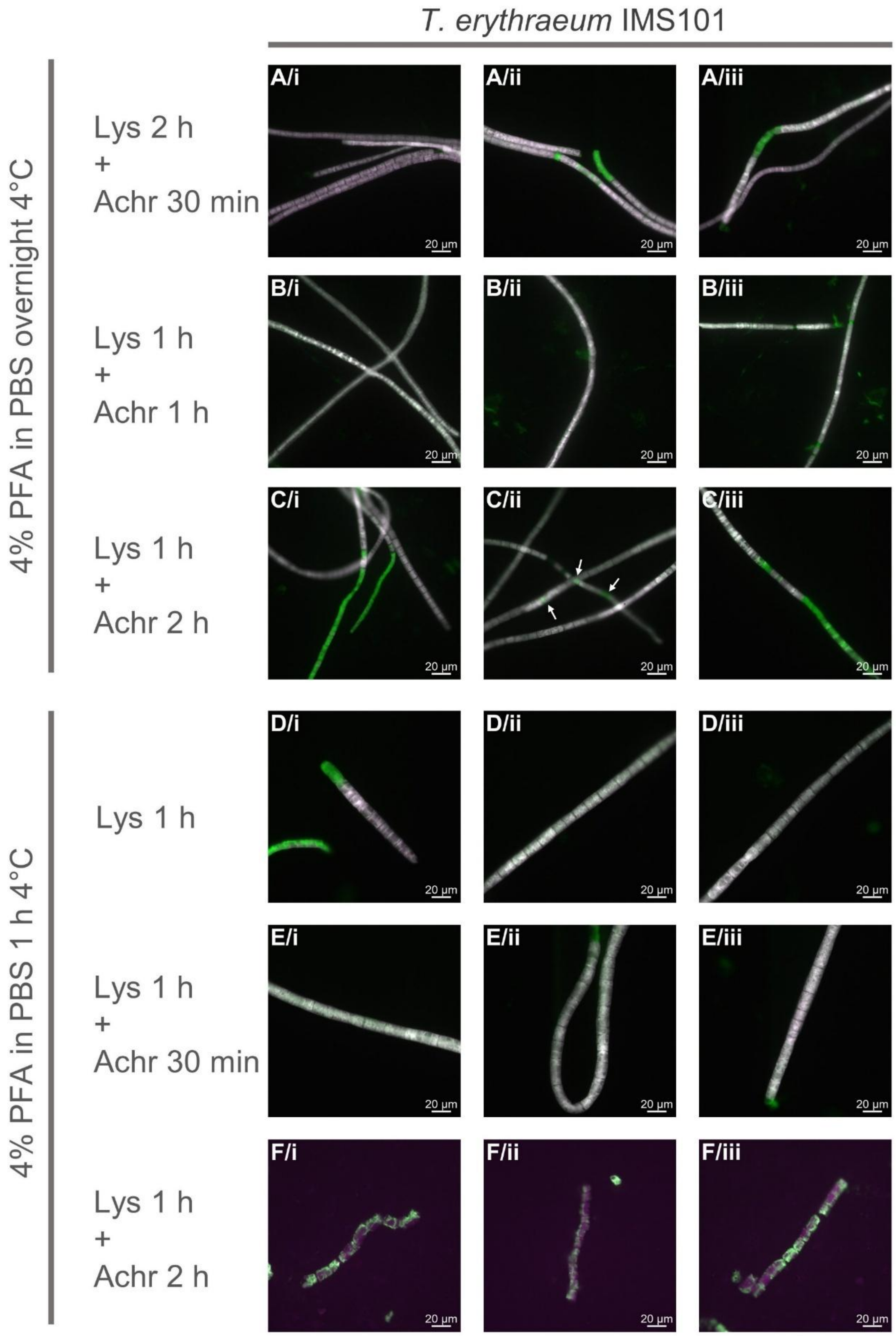

Figure S5. Digestion tests performed on *T. erythraeum* IMS101 strain, related to Steps 3, 5, and 6. Images are shown as a merge of EUB signal (Alexa488, green) and *Trichodesmium*'s autofluorescence (546, pink). Note the improved digestion efficiency during longer incubation time in achromopeptidase compared to lysozyme (C). Each row represents filaments from the same sample. White arrows point to hybridized *Trichodesmium* cells. All scale bars: 20 µm. PFA = paraformaldehyde; Lys = lysozyme; Achr = achromopeptidase. A) 4% PFA in PBS overnight 4°C, Lys 2 h + Achr 30 min. B) 4% PFA in PBS overnight 4°C, Lys 1 h + Achr 1 h. C) 4% PFA in PBS overnight 4°C, Lys 1 h + Achr 2 h. D) 4% PFA in PBS 1 h 4°C, Lys 1 h. E) 4% PFA in PBS 1 h 4°C, Lys 1 h + Achr 30 min. F) 4% PFA in PBS 1 h 4°C, Lys 1 h + Achr 2 h.

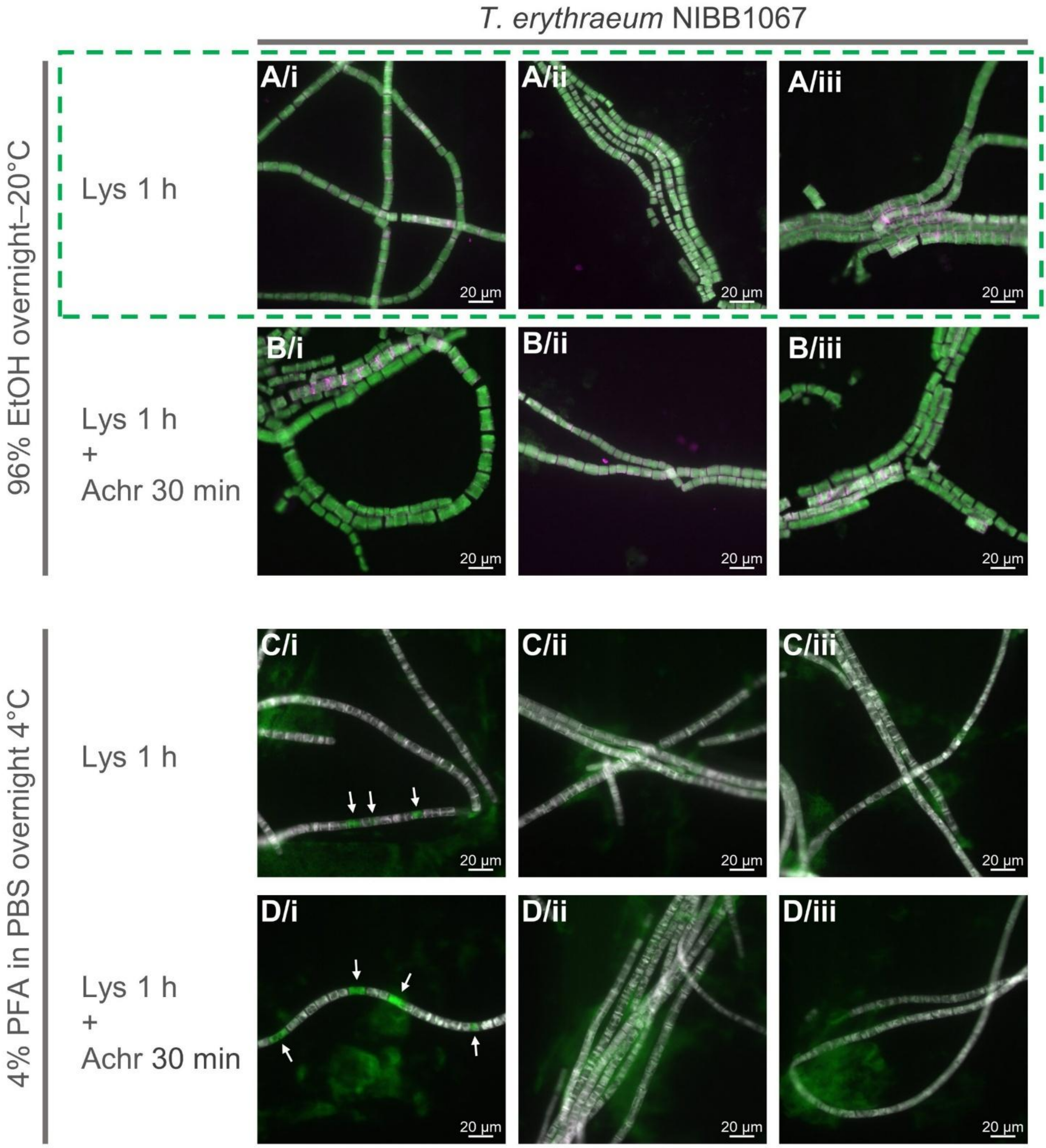

Figure S6. Digestion tests performed on *T. erythraeum* NIBB1067 strain, related to Steps 3, 5, and 6. Images are shown as a merge of EUB signal (Alexa488, green) and *Trichodesmium*'s autofluorescence (546, pink). The green dashed rectangle highlights the optimal fixation and digestion conditions, according to our tests. The first two rows represent filaments from three different samples of three different experiments, while the others represent filaments from the same sample. White arrows point to hybridized *Trichodesmium* cells. All scale bars: 20 µm. EtOH = ethanol; PFA = paraformaldehyde; Lys = lysozyme; Achr = achromopeptidase. A) 96% EtOH overnight −20°C, Lys 1 h. B) 96% EtOH overnight −20°C, Lys 1 h + Achr 30 min. C) 4% PFA in PBS overnight 4°C, Lys 1 h. D) 4% PFA in PBS overnight 4°C, Lys 1 h + Achr 30 min.

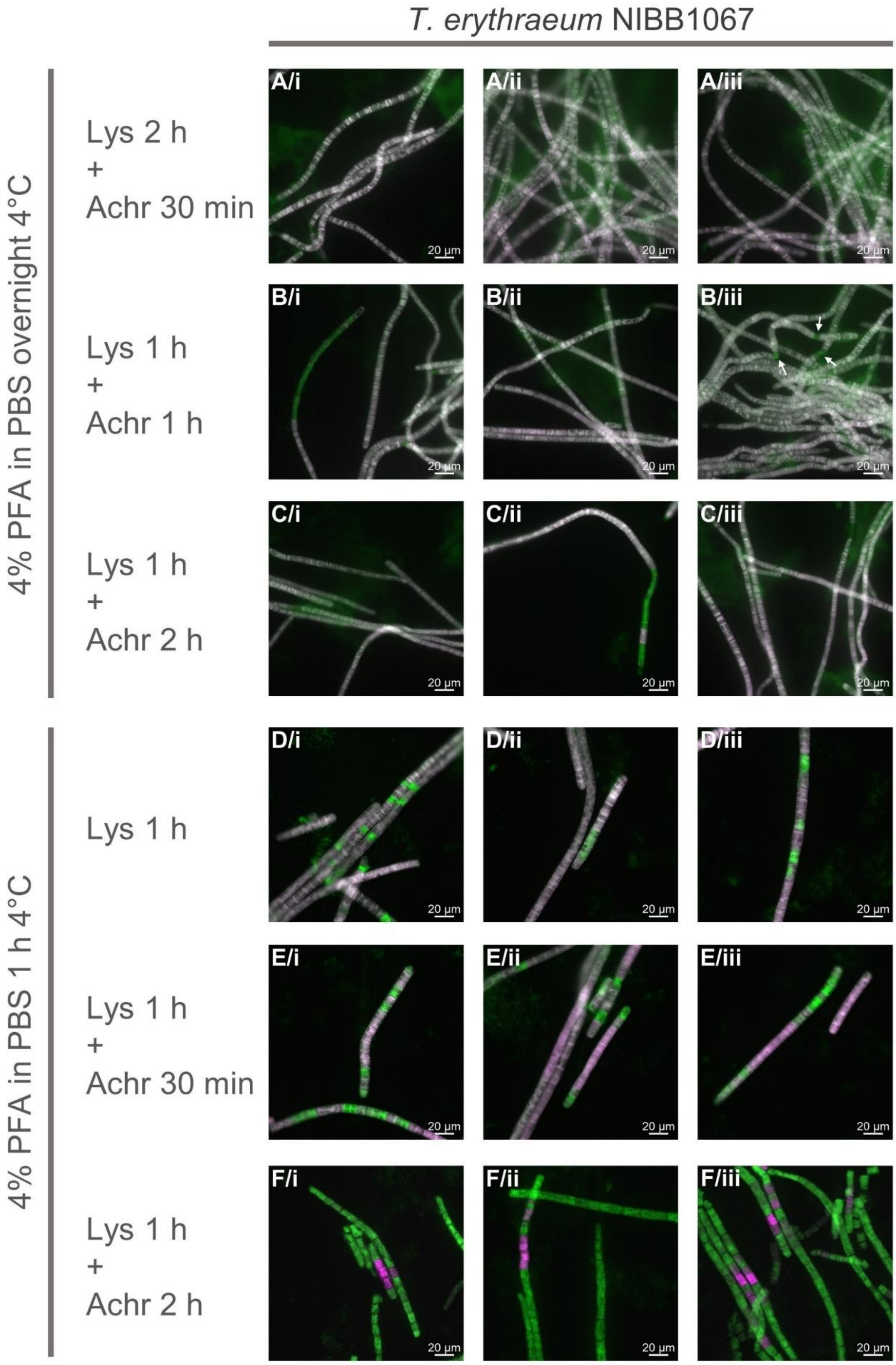

Figure S7. Digestion tests performed on *T. erythraeum* NIBB1067 strain, related to Steps 3, 5, and 6. Images are shown as a merge of EUB signal (Alexa488, green) and *Trichodesmium*'s autofluorescence (546, pink). Note the improved digestion efficiency during longer incubation time in achromopeptidase compared to lysozyme (F). Each row represents filaments from the same sample. White arrows point to hybridized *Trichodesmium* cells. All scale bars: 20 µm. PFA = paraformaldehyde; Lys = lysozyme; Achr = achromopeptidase. A) 4% PFA in PBS overnight 4°C, Lys 2 h + Achr 30 min. B) 4% PFA in PBS overnight 4°C, Lys 1 h + Achr 1 h. C) 4% PFA in PBS overnight 4°C, Lys 1 h + Achr 2 h. D) 4% PFA in PBS 1 h 4°C, Lys 1 h. E) 4% PFA in PBS 1 h 4°C, Lys 1 h + Achr 30 min. F) 4% PFA in PBS 1 h 4°C, Lys 1 h + Achr 2 h.

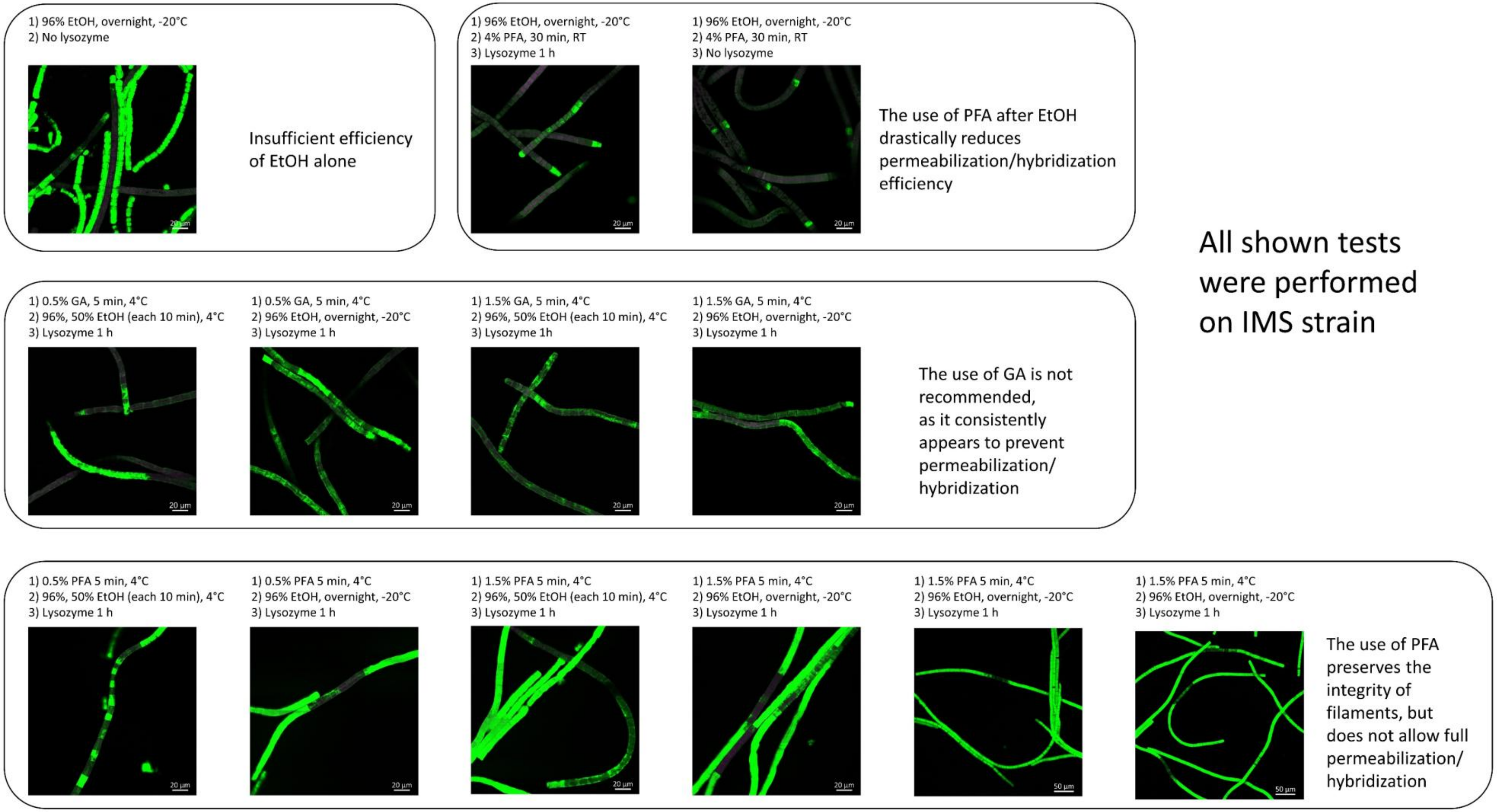

Figure S8. Digestion tests performed on *T. erythraeum* IMS101 strain, related to Steps 3, 5, and 6. Images are shown as a merge of EUB signal (Alexa488, green) and autofluorescence (543, pink). 1) to 3) brief descriptions of the performed steps, and short side notes that explain why the use of PFA/GA is not suitable. All scale bars are 20 µm, except for the two images at bottom right, whose scale bars are 50 µm. EtOH = ethanol; PFA = paraformaldehyde; GA = glutaraldehyde; RT = room temperature.

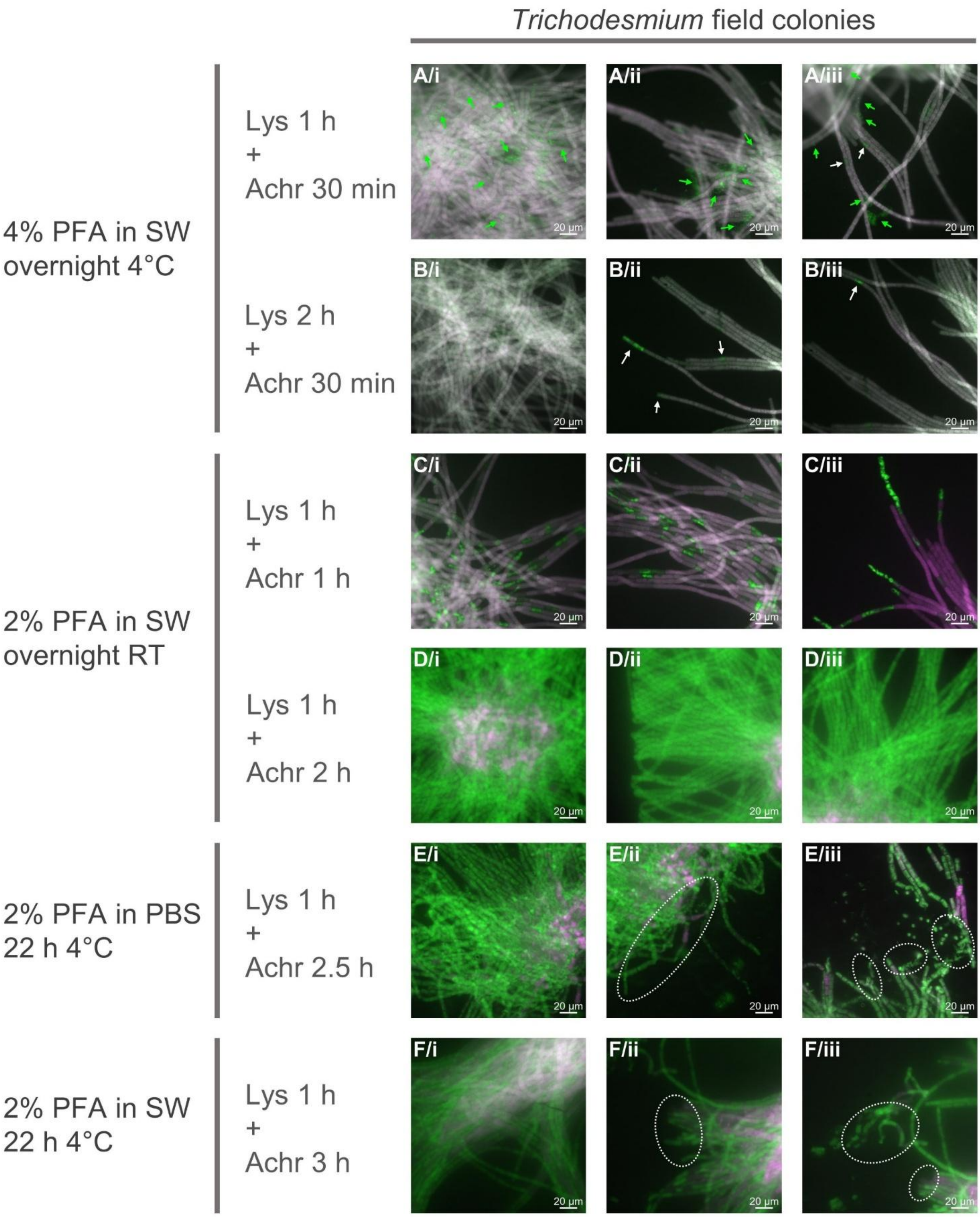

Figure S9. Digestion tests performed on *Trichodesmium* field colonies, related to Steps 3, 5, and 6. Images are shown as a merge of EUB signal (Alexa488, green) and *Trichodesmium*'s autofluorescence (546, pink). Note the improved digestion efficiency during longer incubation time in achromopeptidase compared to lysozyme (D). Each row represents different regions of the same colony. White arrows point to hybridized *Trichodesmium* cells; green arrows indicate the presence of hybridized associated bacteria; dotted circles surround damaged regions. All scale bars: 20 µm. PFA = paraformaldehyde; SW = sea water; RT = room temperature; Lys = lysozyme; Achr = achromopeptidase. A) 4% PFA in SW overnight 4°C, Lys 1 h + Achr 30 min. B) 4% PFA in SW overnight 4°C, Lys 2 h + Achr 30 min. C) 2% PFA in SW overnight RT, Lys 1 h + Achr 1 h. D) 2% PFA in SW overnight RT, Lys 1 h + Achr 2 h. E) 2% PFA in PBS 22 h 4°C, Lys 1 h + Achr 2.5 h. F) 2% PFA in SW 22 h 4°C, Lys 1 h + Achr 3 h.

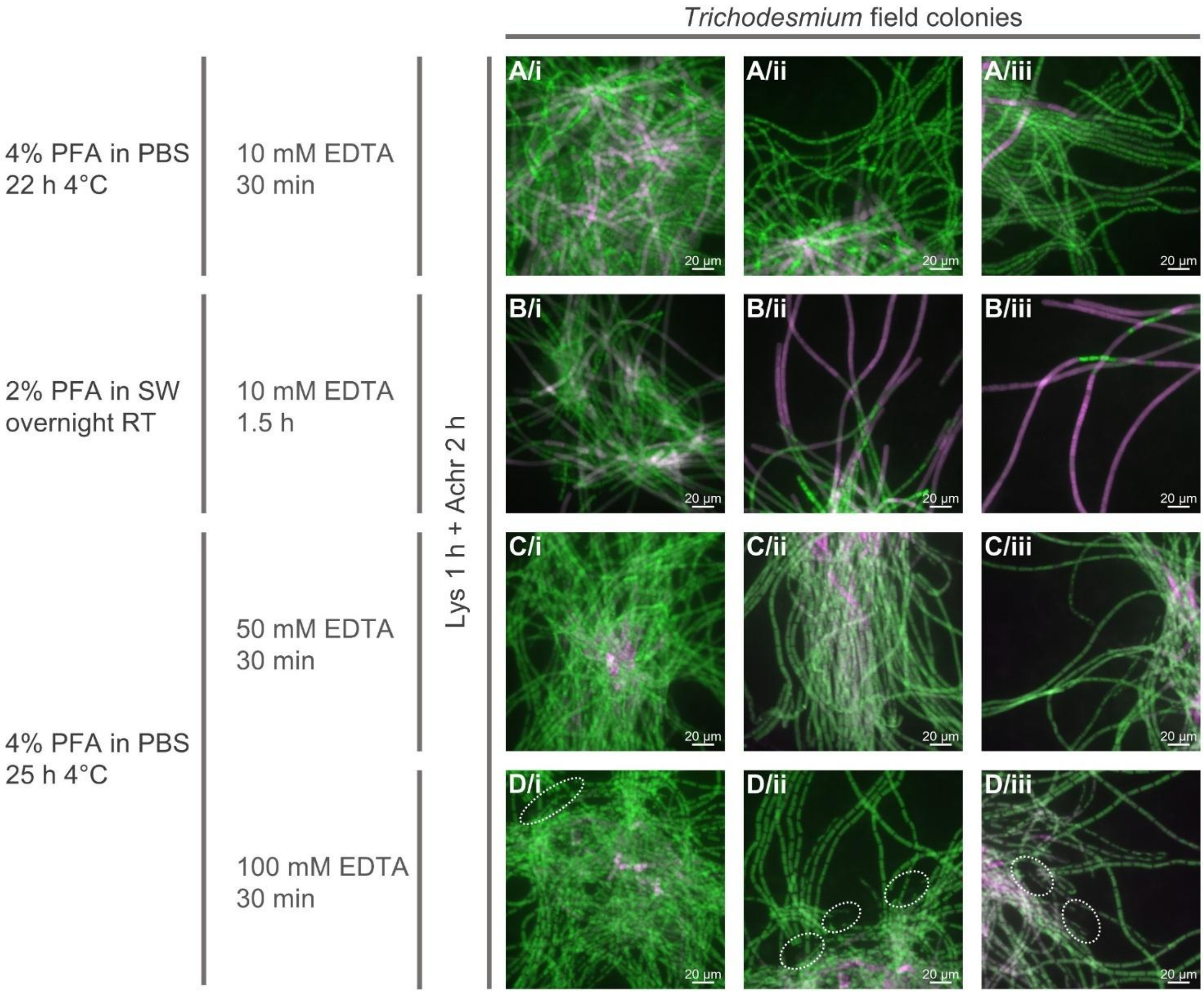

Figure S10. Digestion tests performed on *Trichodesmium* field colonies, related to Steps 3, 5, and 6. Images are shown as a merge of EUB signal (Alexa488, green) and *Trichodesmium*'s autofluorescence (546, pink). Dotted circles surround damaged regions. Each row represents different regions of the same colony. All scale bars: 20 µm. PFA = paraformaldehyde; SW = sea water; RT = room temperature; Lys = lysozyme; Achr = achromopeptidase. A) 4% PFA in PBS 22 h 4°C, 10 mM EDTA 30 min, Lys 1 h + Achr 2 h. B) 2% PFA in SW overnight RT, 10 mM EDTA 1.5 h, Lys 1 h + Achr 2 h. C) 4% PFA in PBS 25 h 4°C, 50 mM EDTA 30 min, Lys 1 h + Achr 2 h. D) 4% PFA in PBS 25 h 4°C, 100 mM EDTA 30 min, Lys 1 h + Achr 2 h.

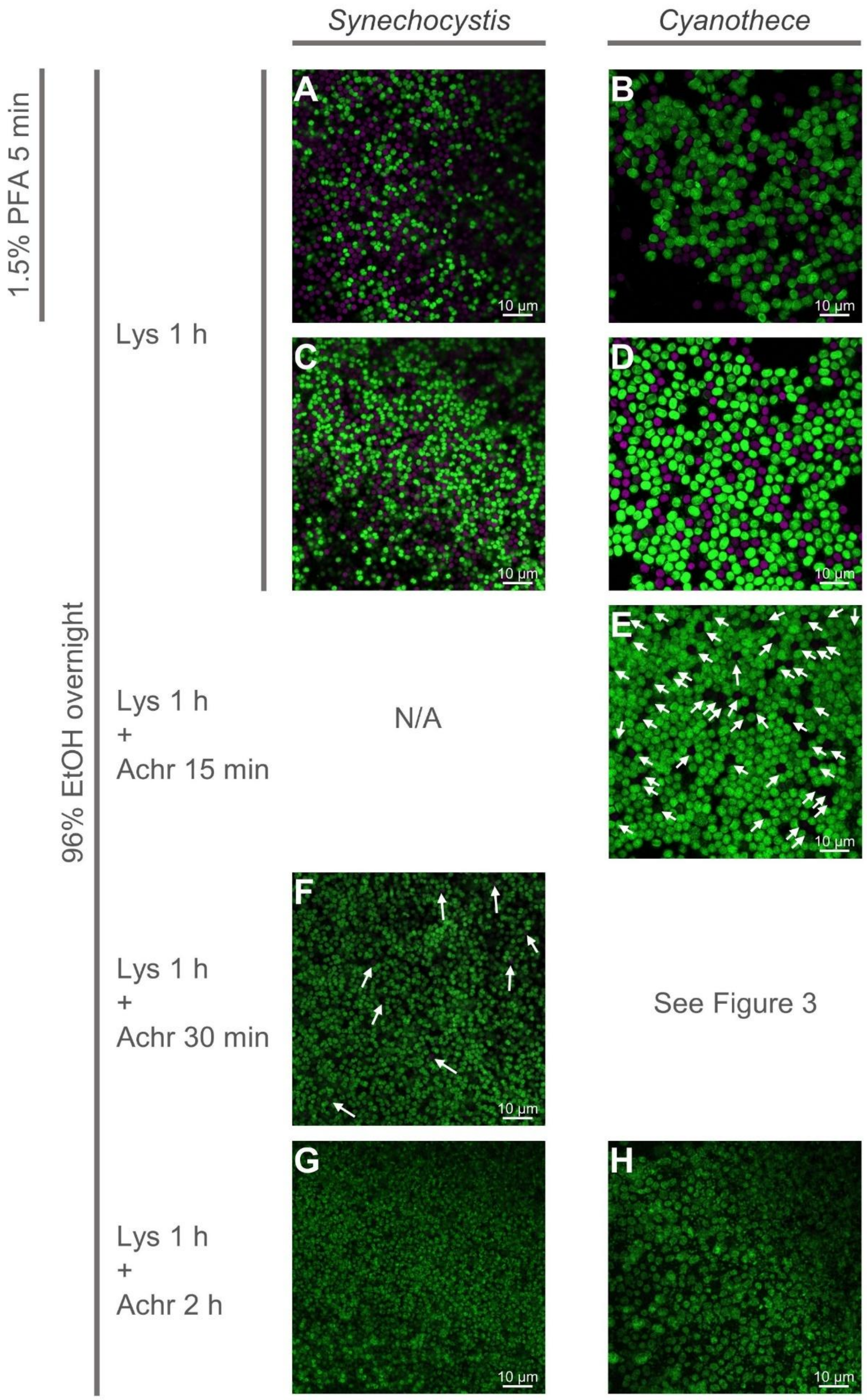

Figure S11. Digestion tests performed on *Synechocystis* and *Cyanothece*, related to Steps 3, 5, and 6. Images are shown as a merge of EUB signal (Alexa488, green) and *Synechocystis* and *Cyanothece* autofluorescence (543, pink). Note overdigestion in panels F–H. White arrows indicate non-labeled cells. All scale bars: 10 µm. EtOH = ethanol; PFA = paraformaldehyde; Lys = lysozyme; Achr = achromopeptidase. A) *Synechocystis*, 1.5% PFA 5 min, 96% EtOH overnight, Lys 1 h. B) *Cyanothece*, 1.5% PFA 5 min, 96% EtOH overnight, Lys 1 h. C) *Synechocystis*, 96% EtOH overnight, Lys 1 h. D) *Cyanothece*, 96% EtOH overnight, Lys 1 h. E) *Cyanothece*, 96% EtOH overnight, Lys 1 h + Achr 15 min. F) *Synechocystis*, 96% EtOH overnight, Lys 1 h + Achr 30 min. G) *Synechocystis*, 96% EtOH overnight, Lys 1 h + Achr 2 h. H) *Cyanothece*, 96% EtOH overnight, Lys 1 h + Achr 2 h.

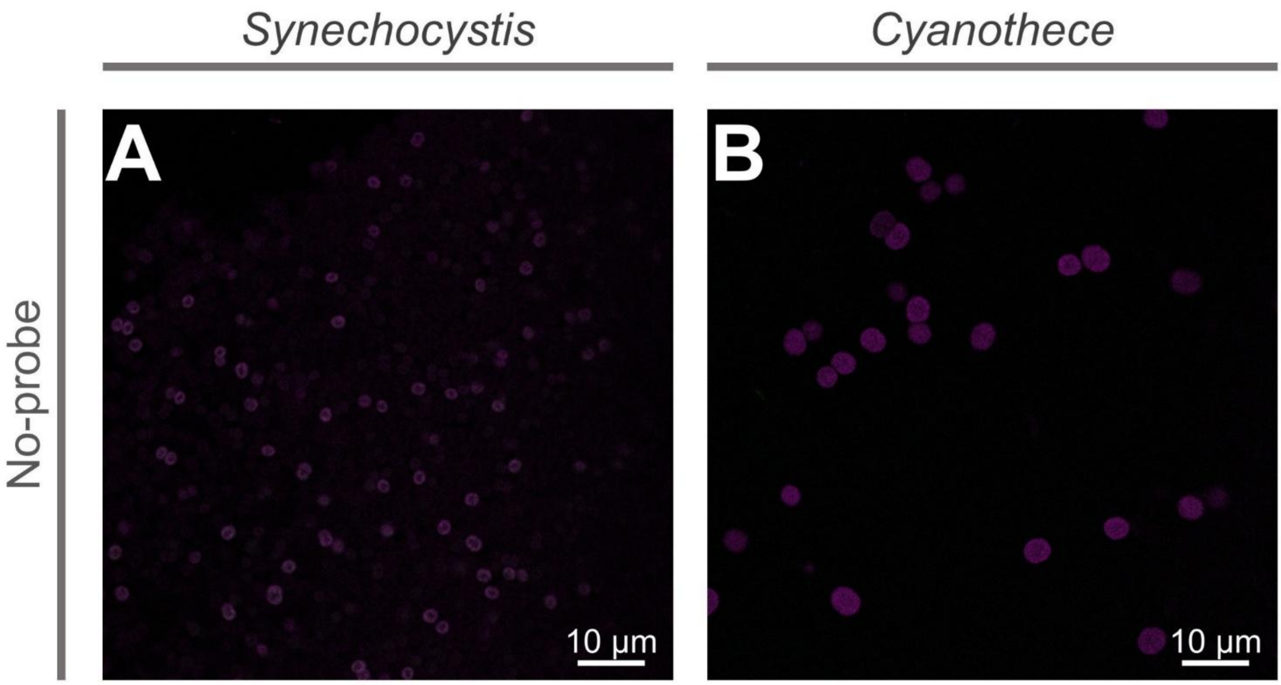

Figure S12. No-probe negative control tested on *Synechocystis* and *Cyanothece*, related to Step 12b. Images are shown as a merge of both autofluorescence signals (Alexa488, green; 543, pink). Both scale bars: 10 µm. A) *Synechocystis*. B) *Cyanothece*.
